# Differentiating the 2D Passivation from Amorphous Passivation in Perovskite Solar Cells

**DOI:** 10.1007/s40820-025-01913-y

**Published:** 2025-09-08

**Authors:** Xiaojian Zheng, Shehzad Ahmed, Yu Zhang, Guoqiang Xu, Junyu Wang, Di Lu, Tingshu Shi, Jun Tang, Lei Yan, Wei Chen, Peigang Han, Zhixin Liu, Danish Khan, Xingzhu Wang, Zeguo Tang

**Affiliations:** 1 College of New Materials and New Energies, Shenzhen Technology University, Lantian Road 3002, Pingshan, 518118 Shenzhen People’s Republic of China; 2 Engineering and Research Center for Integrated New Energy Photovoltaics & Energy Storage Systems of Hunan Province and School of Electrical Engineering, University of South China, Hengyang, 421001 People’s Republic of China; 3 China-UK Low Carbon College, Shanghai Jiao Tong University, Lingang, 201306 Shanghai People’s Republic of China; 4 College of Engineering Physics, Shenzhen Technology University, Shenzhen, 518118 Guangdong People’s Republic of China

**Keywords:** 3D/2D perovskite films, Benzamidine, Amorphous passivation, 2D passivation, Inverted perovskite solar cells

## Abstract

The introduction of two-dimensional (2D) perovskite layers on top of three-dimensional (3D) perovskite films enhances the performance and stability of perovskite solar cells (PSCs). However, the electronic effect of the spacer cation and the quality of the 2D capping layer are critical factors in achieving the required results. In this study, we compared two fluorinated salts: 4-(trifluoromethyl) benzamidine hydrochloride (4TF-BA·HCl) and 4-fluorobenzamidine hydrochloride (4F-BA·HCl) to engineer the 3D/2D perovskite films. Surprisingly, 4F-BA formed a high-performance 3D/2D heterojunction, while 4TF-BA produced an amorphous layer on the perovskite films. Our findings indicate that the balanced intramolecular charge polarization, which leads to effective hydrogen bonding, is more favorable in 4F-BA than in 4TF-BA, promoting the formation of a crystalline 2D perovskite. Nevertheless, 4TF-BA managed to improve efficiency to 24%, surpassing the control device, primarily due to the natural passivation capabilities of benzamidine. Interestingly, the devices based on 4F-BA demonstrated an efficiency exceeding 25% with greater longevity under various storage conditions compared to 4TF-BA-based and the control devices.

**Supplementary Information:**

The online version contains supplementary material available at 10.1007/s40820-025-01913-y.

## Highlights

- Benzamidine derivatives are utilized to differentiate between 2D passivation and amorphous passivation.
- Introducing an n-type 2D passivation layer enhances the charge extraction and transportation and reduces the interface recombination in inverted perovskite solar cells.
- The intramolecular charge of organic ligands is critical for the formation of crystalline 2D capping layers on 3D perovskite layers.
- The long-term stability of inverted perovskite solar cells is improved owing to hydrophobic sealing of 3D perovskite via crystalline 2D capping.

**Supplementary Information:**

The online version contains supplementary material available at 10.1007/s40820-025-01913-y.

## Graphical abstract

**Figure:**
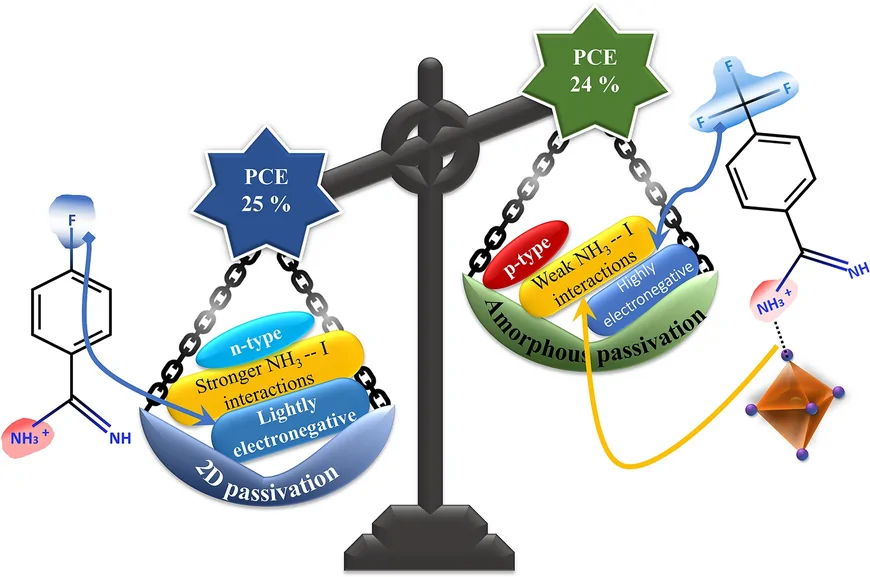

## Introduction

Metal halide perovskite solar cells (PSCs) have emerged as one of the most promising photovoltaic technologies for practical applications. This is primarily due to their combination of relatively low production costs, enabled by solution-based processing techniques, and their superior power conversion efficiencies (PCEs). To date, state-of-the-art single-junction PSCs have achieved certified efficiencies of 27%, which is comparable to the PCE of commercial silicon solar cells [1]. Compared to the n-i-p structure, p-i-n-structured PSCs offer significant advantages for broader applications, such as low-temperature fabrication, excellent operational stability, and scalability for large-area production [2–5]. Their design makes them particularly suitable for fabricating tandem and flexible solar cells, highlighting their diverse potential for future photovoltaic technologies [6, 7]. Recent advancements in the performance of PSCs have been achieved by mitigating trap states via ammonia (NH_3_^+^)-based molecules either on the perovskite film's surface or at the interface with the contact layers through effective passivation techniques [8]. Currently, these passivation modes can be divided into two types. The first is the formation of a defined two-dimensional (2D) perovskite capping on a 3D film [9]. Secondly, ammonium cations can bind to the perovskite surface via A-site vacancies or hydrogen bonding, forming a thin molecular passivation layer on top of 3D perovskite [10]. In the 2D passivation case, the 2D layer has been well-established as an effective protective layer on the surface of 3D perovskite films, providing a barrier against moisture and minimizing ion migration [11–13]. This contributes to a notable enhancement in the overall stability of the films. Nevertheless, there are multiple concerns about the 3D/2D heterojunction films. Does every ammonium cation-based organic ligand convert the residual PbI_2_ to 2D perovskites, and if not, then why? Han et al. deposited the 3,5-difluoro-benzamidine hydrochloride (3,5-DFBH) on 3D perovskite film, and no signs of low-dimensional perovskites were noticed [14]. In contrast, when a closely similar type of organic cation (4-amidinopyridine) is mixed in bulk film, it converts to 3D/2D heterostructure perovskites [15]. Furthermore, multiple research studies have been carried out to compare the steric effects of the organic spacers, where the high polarity is found to be effective in forming 2D perovskites on 3D surfaces [16, 17]. Most of the time, fluorinated terminals, due to their high electronegativity and high hydrophobicity, are considered efficient in forming 3D/2D heterojunctions compared to others [18–24]. However, an exceptionally high electronegativity could impact the electronic effect of forming an effective 3D/2D perovskite heterojunction. For instance, in a famous study, 4-trifluoromethyl-phenylammonium (CF_3_-PA) and 4-fluorophenethylamine (F-PEA) are used to create 3D/2D heterojunctions, where 2D perovskite has only been detected in F-PEA’s case, while it remained unobserved in CF_3_-PA’s case [25]. Lin et al. also observed that the CF_3_-blessed PEA cation was unable to form a 2D perovskite film [26]. A couple of other 3D/2D heterostructures have been discovered in which the CF_3_ at the para-position launches a crystalline 2D perovskite rather than at the meta-position at the ring of the conjugated cation [27, 28]. Therefore, we believe that intramolecular charge balance and the electronic effect of the organic cation may influence the formation of 2D perovskite. Furthermore, the presence of amidine groups in the organic spacer can conduct robust hydrogen bonding as compared to conventional amines owing to its nitrogen–carbon–nitrogen backbone (R–C(= NH)-NH_2_), as the properties of both the imine and amine functionalities are available [29–32]. Therefore, amidinio-based ligands are considered effective passivating agents when used as surface passivation or in the formation of 2D perovskites at the surface of 3D perovskite or in bulk [21–24].

Herein, we chose two similar types of spacer cations to construct 3D/2D heterojunctions. One is lightly fluorinated, and the other one is highly fluorinated, i.e., 4-(trifluoromethyl)benzamidine (4TF-BA) and 4-fluorobenzamidine (4F-BA), respectively. 4TF-BA is a bulky and highly electron-withdrawing trifluoromethyl (–CF₃) group, which introduces steric hindrance and alters the electronic environment of the amidine moiety, while 4F-BA has a smaller and less electron-withdrawing –F substituent. We deposited these two salts (4TF-BA·HCl and 4F-BA·HCl) on the perovskite films, which are converted to protonated form (NH_2_ to NH_3_^+^) after dissolving in solvent and can form 2D perovskites [33]. Surprisingly, less fluorinated 4F-BA forms highly crystalline and well-oriented 2D capping for the 3D perovskite layer. On the flip side, 4TF-BA failed to convert to 2D perovskite fully and transformed into an amorphous layer as observed in the scanning electron microscopy (SEM), grazing-incidence wide-angle X-ray scattering (GIWAXS), X-ray diffraction (XRD), and atomic force microscopy (AFM) analysis. We further realized that the moderate electron-withdrawing property of fluorine at the terminal 4F-BA is more beneficial than the extra-high electron-withdrawing property of CF_3_, which launches the electron-deficient environment in the conjugated ring of 4TF-BA. Our theoretical results support these findings. As a result, the well-oriented and defined formation of 4F-BA-based 3D/2D films reduces the work function of perovskite films, while the passivation by 4TF-BA further enhances the work function of perovskite films. In other words, we could say that the 4F-BA film is likely n-type and a good fit for p-i-n structures, thus enhancing the charge extraction and transportation properties in p-i-n PSCs. By clearing the curiosity of 2D formation in one case only, we performed a comprehensive theoretical analysis and realized that the imbalanced Löwdin charge distribution within the molecular structure of 4TF-BA causes incomplete 2D perovskite formation, which further leads to low formation energies of 4TF-BA-based 2D perovskite and low interaction between 4TF-BA and PbI_6_ layer. Consequently, 4F-BA-based devices achieved a PCE of 25.02% with a significantly enhanced fill factor (*FF*) of 84.5%, while 4TF-BA achieved an efficiency of 24.01%. In addition, the unencapsulated 4F-BA-based PSCs delivered longer life spans than 4TF-BA-based devices under highly humid and high-temperature environments.

## Experimental Section

### Materials

All chemicals were used as received without any further purification. N, N-Dimethylformamide (DMF, anhydrous, 99.8%), dimethyl sulfoxide (DMSO, anhydrous, ≥ 99.9%), chlorobenzene (CB, anhydrous, 99.8%), bathocuproine (BCP, 96.0%), and isopropyl alcohol (IPA) were bought from Sigma-Aldrich. Phenyl-C61-butyric acid methyl ester (PC_61_BM) and NiO_X_ nanoparticles were procured from Advanced Election Technology. Methylammonium chloride (MACl, 99.9%), methylammonium iodide (MAI, 99.9%), formamidinium iodide (FAI, 99.99%), cesium iodide (CsI, 99.9%), and lead iodide (PbI_2_, 99.999%) were received from Xi'an Shuo yuan Optoelectronic Technology Co. Ltd. [4-(3,6-Dimethyl-9H-carbazol-9-yl) butyl] phosphonic acid (Me-4PACz), 98%) was gained from TCI. Magnesium fluoride (MgF_2_, 98.0%), 4TF-BA, and 4F-BA were acquired from Shanghai Aladdin Biochemical Technology Co., Ltd.

### Device Fabrication

All of the devices were prepared on FTO-coated glass substrates with dimensions of 2.45 × 2.45 × 0.2 cm and a sheet resistance of 10 Ω sq^−1^. The substrates were sequentially cleaned by sonicating in deionized water (DIW), isopropanol, and ethanol for 25 min at room temperature, followed by drying with nitrogen gas, and then treated with UV-ozone for 15 min to remove any residual chemicals and promote the surface hydrophobicity of the FTO substrates. The NiO_x_ dispersion was prepared by mixing the powder in DIW at a concentration of 20 mg mL^−1^. The solution was then spin-coated onto an FTO substrate at 2000 r min^−1^ for 30 s. Finally, the deposited film was thermally annealed in ambient air at 150 °C for 30 min. After cooling to room temperature, the substrate was transferred into a nitrogen-purged glovebox. Subsequently, a solution of Me-4PACz (0.5 mg mL^−1^ in IPA) was deposited via spin coating at 4000 r min^−1^ for 30 s, followed by thermal annealing at 100 °C for 10 min. The perovskite precursor solution with a composition of FA_0.85_MA _0.1_Cs_0.05_PbI_3_ was prepared by dissolving 23.84 mg MAI,12.15 mg MACl, 219.27 mg FAI, 19.48 mg CsI, and 753.75 mg PbI_2_ in a 1-mL mixed solvent of DMF and DMSO (4:1 v/v), yielding a 1.5-M stoichiometric solution. The perovskite films were prepared by spin-coating using a two-step process, i.e., an initial step at 1000 r min^−1^ for 10 s followed by a second step at 5000 r min^−1^ for 40 s. During the second step, 160 μL of CB was rapidly added to the rotating substrate 5 s before the end of the perovskite film fabrication process. The as-deposited films were then thermally annealed at 100 °C for 30 min in the glove box. For passivation, upon cooling, solutions of 4TF-BA·HCl and 4F-BA·HCl were prepared in IPA with a concentration of 1.5 mg/mL and were spin-coated onto the perovskite layer at 4000 r min^−1^ for 30 s, respectively. This surface treatment was completed after thermal annealing at 100 °C for 10 min. The electron transport layer and the hole-blocking layer were sequentially spin-coated with PC61BM (20 mg mL^−1^ in CB) and BCP (0.5 mg mL^−1^ in IPA) solutions, respectively. Finally, 100 nm of Ag was thermally evaporated as a back contact, and 100 nm of MgF_2_ was thermally evaporated on the glass side as an antireflection coating.

### Characterization

XRD patterns were characterized on a Rigaku SmartLab diffractometer equipped with a CuKα radiation source (λ = 1.5405 Å). GIWAXS measurements were implemented on an in-house Ganesha SAXSLAB instrument with the X-ray photon energy of 8.05 keV and a Pilatus 300 K detector. The incidence angle was set at 0.4°, and the sample detector distance was 95 mm. XPS analysis was performed on a Thermo Scientific™ ESCALAB 250Xi system equipped with a monochromatic Al Kα X-ray source (hν = 1,486.6 eV). SEM surface and cross-sectional images were obtained on a ZEISS GeminiSEM 300 field-emission microscope. AFM surface morphology and work function mapping were obtained using a Bruker Dimension Icon system in PeakForce KPFM mode. Absorption spectra were recorded on a Jasco UV-2600 UV–Vis spectrophotometer. The contact angles were acquired on a Kruss DSA100 contact angle goniometer. Steady-state photoluminescence (PL) spectra and time-resolved photoluminescence (TRPL) spectra were characterized on a fluorescence lifetime testing system (FluoTime 300, Picoquant). The current density–voltage (*J–V*) curves of the devices under AM1.5G illumination and dark conditions were recorded using a solar simulator connected to a Keithley 2400 digital source meter. The light intensity calibration was carried out using a standard silicon photodiode, and the scan rate was set to 20 mV s^−1^, while the dwell time was fixed at 0.01 s. External quantum efficiency (EQE) spectra were evaluated using monochromatic illumination (Oriel Cornerstone260 1/4 m monochromator with an Oriel 70613NS QTH lamp from Enlitech). Electrochemical impedance spectroscopy (EIS) measurements were implemented on an electrochemical workstation (PAR-Ametek, VersaSTAT 3).

## Results and Discussion

**Fig. 1:**
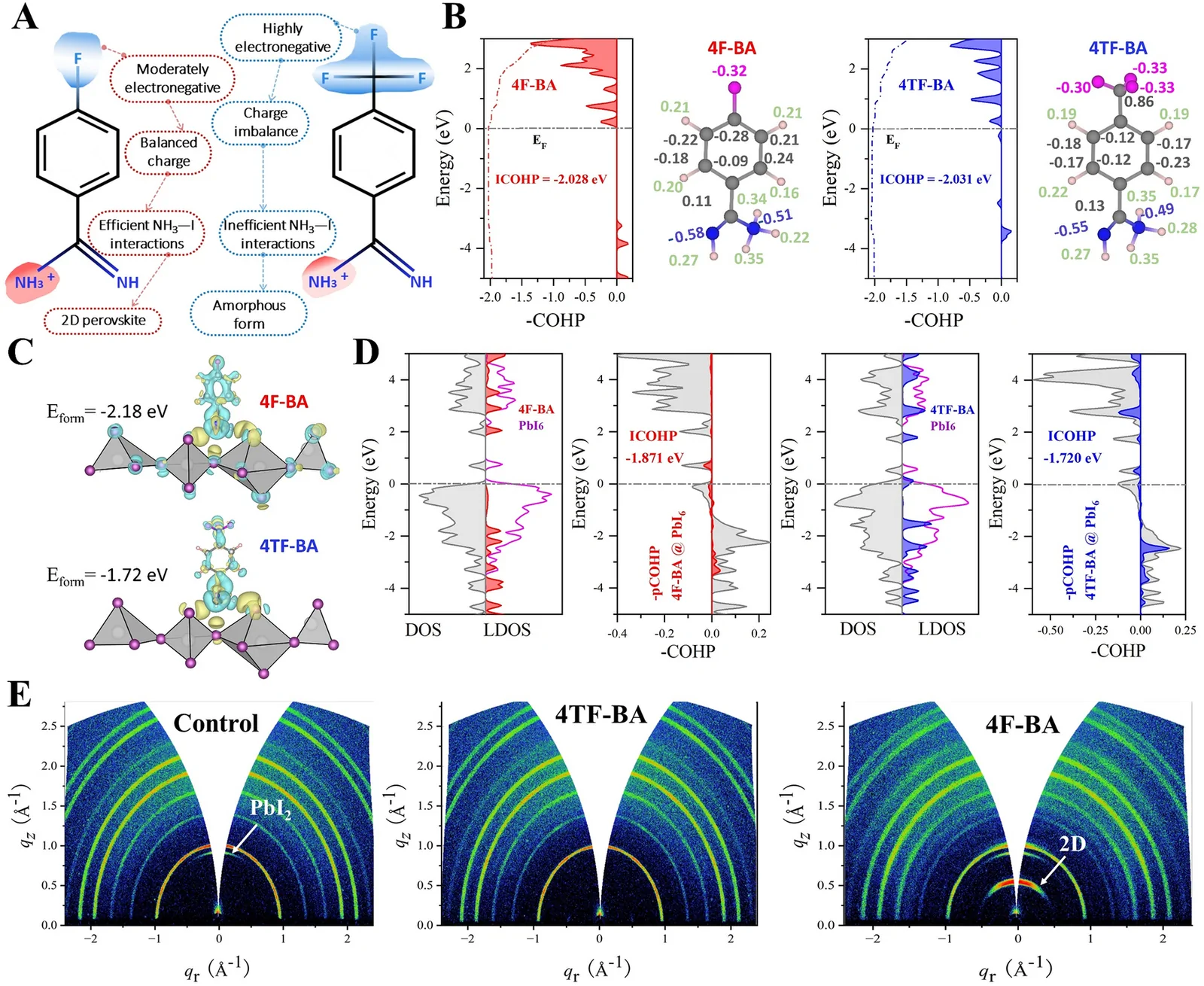
**A** The schematic representation and chemical structures of 4F-BA and 4TF-BA. **B** Chemical bonding insight -COHP, ICOHP bonding strength analysis, and corresponding Löwdin charges on 4F-BA and 4TF-BA. **C** The formation energies after chemical adsorption and charge transfer isosurface. **D** Density of states (DOS), Local DOS of adsorbed molecules and PbI_6_ layer, and chemical bonding -COHP, -pCOHP, including bonding strength ICOHP analysis of 4F-BA and 4TF-BA adsorbed on the PbI_6_ layer. **E** GIWAXS images of control, 4TF-BA-based, and 4F-BA-based films

The residual PbI_2_ in the perovskite film is transformed into a stable and crystalline 2D perovskite upon the deposition of 4F-BA·HCl. However, incomplete perovskite phases or amorphous films appear when 4TF-BA·HCl is applied to the 3D perovskite film. From multiple theoretical analyses, we realized that the high electron-withdrawing capability of CF_3_ introduces the uneven charge distribution in 4TF-BA’s case, further reducing the NH_3_···I interactions. On the flip side, the balanced charge distribution of 4F-BA helps in executing stable NH_3_···I interactions, as shown in the schematic diagram of Fig. 1A. The crystal orbital Hamilton population (COHP) technique offers a refined approach to partitioning the density of states (DOS) into bonding and antibonding contributions. Notably, an increased presence of antibonding states at the Fermi level (E_F_) is viewed as a destabilizing factor, highlighting the intricate balance of interactions within a material's electronic structure [34]. Figure 1B presents the integration of COHP (ICOHP) analysis and Löwdin charge distribution of both cations, revealing a stable interaction at the E_F_ with no significant bonding states below. The ICOHP value of -2.028 eV confirms its electronic stability. In contrast, 4TF-BA exhibits a slight antibonding peak below the E_F_ and a marginally lower ICOHP value of -2.031 eV, indicating reduced stability [35, 36]. From the Löwdin charge distribution (Fig. 1B), the nitrogen atom in the NH_3_^+^ group of 4F-BA carries a Löwdin charge of –0.51 e, indicating a relatively higher electron density and thus more electronegative character. In contrast, the nitrogen atom in 4TF-BA exhibits a less negative Löwdin charge of –0.49 e, reflecting a decrease in electron density and an increase in electropositive character. This shift is consistent with the strong electron-withdrawing nature of the CF₃ group, which draws electron density away from the NH_3_^+^ group. On the flip side, in 4F-BA, the comparatively lesser withdrawal by F does not induce significant delocalization, leaving the nitrogen of NH_3_ moderately positive. A similar scenario has been seen in the charge transfer calculations (Fig. 1C). The 4F-BA’s superior performance lies in its molecular design; fluorine atoms selectively withdraw lesser electron density from the carbon ring, localizing negative charge more effectively. From another perspective, the 4F-BA exhibits an n-type behavior, as indicated by the charge loss around the 4F-BA molecules. This charge loss suggests that the layer is electron-rich, thereby increasing the electron density in the surrounding structure. On the other hand, the 4TF-BA layer exhibits higher charge gain and lower charge loss, indicating that it is electron-deficient as compared to 4F-BA. Moreover, 4F-BA exhibits a lowest unoccupied molecular orbital (LUMO) level of -1.06 eV, indicating that it is an electron-deficient material and thus favors electron acceptance and conduction (n-type), as shown in Figure S1. Conversely, the electron-withdrawing CF_3_ group in 4TF-BA creates an electron-deficient environment (p-type) in the benzene ring, raising the LUMO level to -2.07 eV and making it prone to electron donation. Furthermore, the spatial distribution of the LUMO offers critical insight into the origin of the enhanced stability observed in the 4F-BA-based perovskite. In the case of 4F-BA, the LUMO is predominantly localized on the NH_2_^+^ moiety, which serves as the primary interaction site with the inorganic PbI₆ octahedra (Fig. S1). This focused electron-accepting character facilitates stronger directional coupling between the organic cation and the PbI_6_ framework, thereby reinforcing the NH_3_···I interactions that are essential for maintaining structural integrity. Conversely, in 4TF-BA, the LUMO is delocalized across the aromatic ring as a consequence of the strong electron-withdrawing effect of the CF₃ group. Although NH_3_^+^ of 4TF-BA is more electropositive, a balanced distribution of charges is more favorable for forming 2D perovskite compared to an imbalanced charge distribution. This balanced charge distribution enhances both the formation energy (-2.181 eV) and hydrophobic stabilization at the PbI_6_ interface, creating a robust, multi-mechanism shield. In contrast, 4TF-BA’s fluorine behaves differently, pulling more electron density away from the ring, resulting in weaker interactions (-1.724 eV) and relying solely on NH_3_···I bonds for passivation. The electronic interactions at the spacer cation/PbI_6_ interface were analyzed through Local DOS (LDOS) comparisons between isolated and interacting components [37]. Figure 1D reveals that both molecules exhibit minimal electronic states at the E_F_, with a clear band gap separating the occupied valence states below E_F_ from unoccupied conduction states above. This indicates effective defect passivation in both systems. However, key differences emerge in the -COHP analysis: while 4TF-BA shows an antibonding peak at E_F_ that could promote instability, 4F-BA demonstrates superior stability through stronger bonding interactions. The pCOHP calculations confirm that neither compound exhibits significant Fermi-level interactions, but the integrated-COHP (ICOHP) values reveal substantially stronger bonding for 4F-BA (-1.871 eV) compared to 4TF-BA (-1.720 eV). These results collectively demonstrate that 4F-BA achieves enhanced electronic stability and interfacial charge transfer through more favorable orbital hybridization with the PbI_6_ framework, explaining its superior experimental performance. We also confirmed these findings via Fourier transform infrared spectroscopy (FTIR) (Fig. S2). The observed red shift of the N–H stretching vibration from 3418.1 to 3412.3 cm⁻^1^ after 2D perovskite surface treatment indicates an enhancement in hydrogen bonding strength between the N–H groups of the 2D cations and the iodide ions of PbI_6_. This shift to lower wavenumbers is attributed to the weakening of the N–H bond caused by the formation of hydrogen bonds, which reduces the vibrational frequency. Such a red shift is consistent with stronger NH···I interactions, suggesting improved surface passivation and interfacial coupling upon 4F-BA-based 2D layer incorporation. However, in the 4TF-BA case, the reduction and broadening of the N–H stretching peak suggest that the passivating molecules do not assemble into an ordered 2D perovskite phase but instead form an amorphous or disordered surface layer, i.e., reduced NH···I interactions.

To gain further insight into the bonding mechanism and thermal stability, we conducted ab initio molecular dynamics (AIMD) simulations at 300 K and analyzed the structural snapshots taken before and after thermal equilibration (Fig. S3). The bond length evolution of key interfacial and intramolecular interactions shows that 4F-BA approaches the adsorption site more closely than 4TF-BA, indicating stronger binding. At 300 K, even a visual inspection of the structural configuration indicates the high stability of the 2D perovskite formed using 4F-BA as the spacer cation. However, a more rigorous evaluation of structural stability can be performed by analyzing the bond lengths and their variation under AIMD simulations at 300 K. Among the key interactions, two bond types are particularly critical: the hydrogen–iodine (H···I) interaction between the ammonium group (–NH₃⁺) and the iodide of the PbI₆ octahedra, and the Pb–I bonds that define the backbone of the 2D perovskite lattice. Again, the NH₃⁺···I⁻ interaction is significantly stronger in the case of 4F-BA. Supporting this, at 0 K, the H···I bond length is shorter in the 4F-BA-based perovskite compared to that based on 4TF-BA. After AIMD simulations at 300 K, a notable increase in H···I bond length is observed for the 4TF-BA system, whereas a reduction is observed for the 4F-BA-based perovskite. This contrasting trend further confirms the stronger and more stable NH_3_···I interaction in the 4F-BA case. A similar trend is observed in the N–I interactions, providing additional support for this conclusion. Furthermore, the Pb–I bond length, which is critical in maintaining the structural integrity of the 2D perovskite framework, is consistently longer in the 4TF-BA system than in the 4F-BA counterpart. This observation further substantiates the superior structural stability of the 4F-BA-based 2D perovskite at room temperature. Moreover, to evaluate the electronic interactions at the interface between the organic cation and the inorganic PbI₆ framework, we performed a charge gain/loss analysis (Fig. S4). The results reveal that the 4F-BA-based perovskite exhibits more pronounced charge transfer activity compared to its 4TF-BA counterpart. This enhanced interfacial interaction is likely attributable to the stronger electronic coupling between the 4F-BA layer and the PbI₆ octahedral network.

As expected, the GIWAXS and XRD results clearly indicate that only 4F-BA could launch stable and crystalline 2D structures on a 3D perovskite layer. A distinguished and intense peak (at 7.18°) can be seen in the XRD graphs of 4F-BA-based films (Fig. S5), while only a small bump appeared in 4TF-BA’s case. Similar results are extracted from the GIWAXS analysis, i.e., a stronger peak related to the low-dimensional perovskite appearing in 4F-BA-based 3D/2D films (Fig. S6). Accordingly, a sliced and bright ring appeared (at the top of the beam) in the corresponding images of GIWAXS in 4F-BA-based 3D/2D films, indicating the presence of crystalline 2D perovskite structures on 3D films (Fig. 1E) [38]. On the other hand, a faint 2D perovskite ring is observed in 4TF-BA-based films, indicating the incomplete form of 2D perovskites (a zoomed-in version is provided in Fig. S7 for better visualization). Interestingly, the PbI_2_ peak disappeared in 4TF-BA’s case, while 4F-BA-based 3D/2D films still had this peak. We doubt that this is due to the presence of some remaining unreacted PbI_2_ in the bulk or at the interface between the 2D and 3D phases. PbI_2_’s peak disappeared in the case of 4TF-BA, which strengthened the fact of low formation energies and low interactions with the PbI_6_ layer as obtained in our theoretical calculation. In the 4TF-BA case, PbI₂ is absent due to weaker NH₃···I interactions that allow full incorporation into the amorphous 2D perovskite structures [39]. We further designed as pure 2D Ruddlesden–Popper perovskite film (4F-BA)₂Pb(ICl)₄ by mixing 4-fluorobenzamidine hydrochloride (4F-BA·HCl) and PbI₂ in a 2:1 molar ratio, followed by spin-coating onto FTO substrates. The resulting films were annealed at 100 °C for 10 min. As shown in Fig. S8A, scanning electron microscopy (SEM) reveals a crystalline surface morphology. Corresponding XRD patterns (Fig. S8B) exhibit a distinct peak at 7.19°, which closely aligns with those observed in 3D/2D perovskite heterostructures, indicating the formation of a layered perovskite phase.

**Fig. 2:**
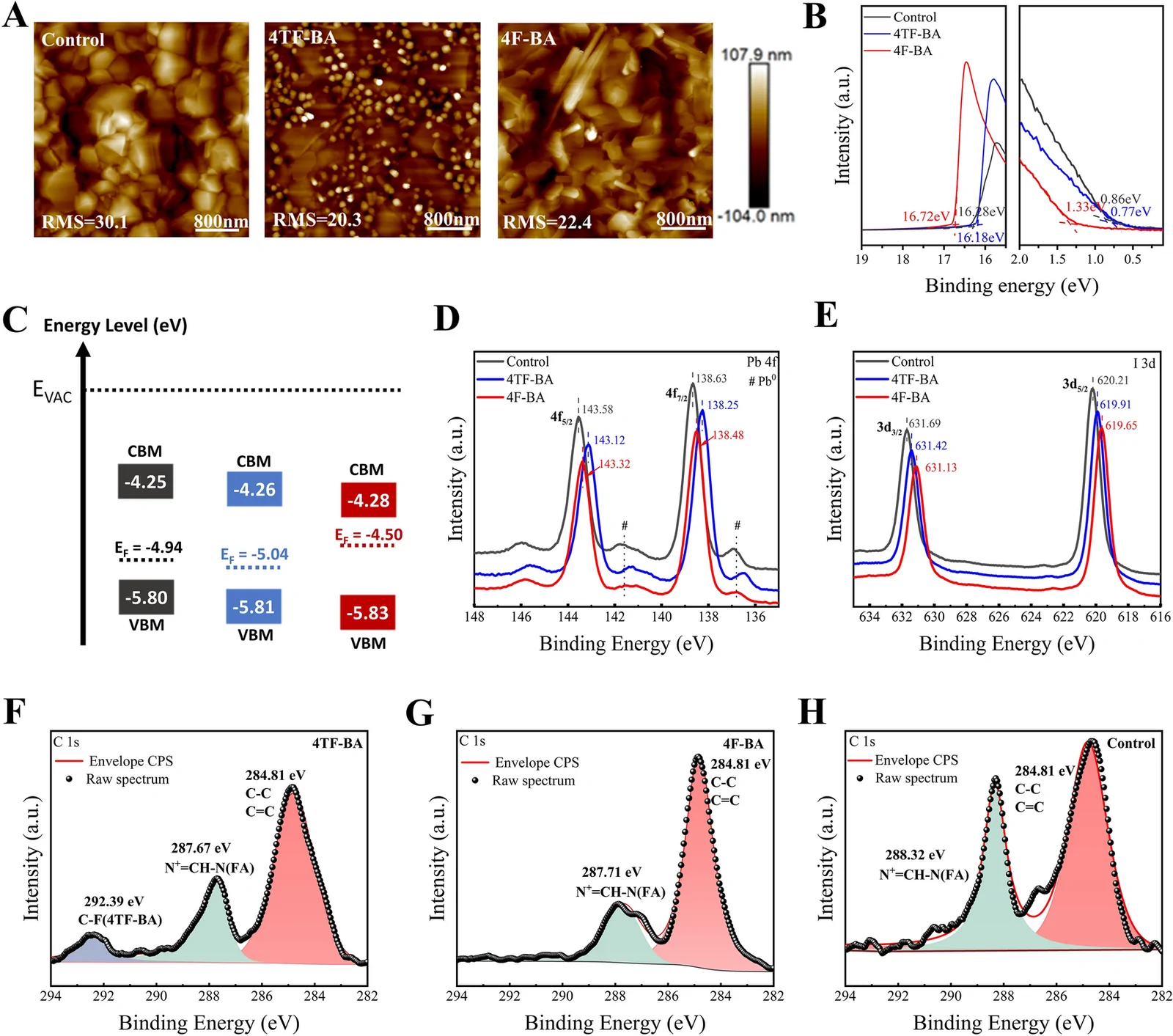
**A** AFM images, **B** ultraviolet photoelectron spectroscopy (UPS) spectra, **C** energy levels and work functions of control, 4TF-BA-treated, and 4F-BA-treated films. XPS spectra of the **D** Pb 4*f*, **E** I 3*d*, and **F–H** C 1*s* regions for the control, 4TF-BA, and 4F-BA-treated perovskite films

SEM images further confirm this fact, as the films based on 4TF-BA exhibit smaller disordered crystallites (Fig. S9), indicating the presence of amorphous or degraded phases. Conversely, control films show the PbI_2_ residue, whereas 4F-BA-based films display the crystalline 2D capping of 3D films. Cross-sectional SEM images further confirm this observation (Fig. S10). Similar amorphous phases for 4TF-BA’s case can also be noticed in AFM images (Fig. 2A), while 4F-BA-based 3D/2D films exhibited a defined shape of 2D perovskites. The root-mean-square (RMS) roughness values were lower in both the modified devices, with slightly lowered values in 4TF-BA’s case. The RMS surface potential, as measured by KPFM, decreased from 14.1 (control) to 12.4 and 11.9 mV for the 4TF-BA and 4F-BA 2D-treated films, respectively (Fig. S11). The observed trend suggests a lower degree of local surface potential fluctuations, which is consistent with improved interfacial passivation and reduced electronic disorder. Additionally, to investigate the changes in built-in potential (*V*_*bi*_)—an important parameter directly linked to the interfacial electric field—we conducted capacitance–voltage (C–V) measurements, as shown in Fig. S12. Based on the Mott–Schottky analysis, the extracted *V*_*bi*_ values for the control, 4TF-BA based, and 4F-BA-based devices were 0.72, 0.85, and 0.94 V, respectively. As extracted from the ultraviolet photoelectron spectroscopy (UPS) and ultraviolet–visible (UV–vis) measurements (Figs. 2B and S13), the bandgap stayed the same in each case, i.e., at 1.55 eV, the conduction band minimum (CBM) shifted nearer to the CBM of PC_61_BM in 4F-BA’s case (Fig. S14). The low energy difference between the E_F_ of 4F-BA perovskite and its CBM further indicates that the 4F-BA establishes an n-type 3D/2D film, while the opposite is the case for the amorphous film. Interestingly, the work function of the 3D/2D films is reduced for 4F-BA films, while it is enhanced in the case of amorphous film (for 4TF-BA), as shown in Fig. 2C. At this point, we could realize the importance of an n-type 2D capping on the 3D films, which enhances the electron extraction and reduces the recombination paths by reducing the Fermi levels of the absorber film. From the Fermi levels (Fig. 2C), we can conclude that the amorphous film based on 4TF-BA was p-type, while the 4F-BA made the film more n-type, which is beneficial for p-i-n structures. Furthermore, films incorporating the amorphous form of 4TF-BA initially exhibit enhanced absorption, which can be attributed to their disordered structure and increased diffuse light scattering. This observation is supported by SEM and AFM analyses, where amorphous regions and scattered surface features are clearly visible. However, this enhanced optical behavior is not stable over time—after ambient aging, a noticeable degradation in absorption occurs. This further substantiates the presence of an unstable amorphous layer in the 4TF-BA system, in contrast to the more stable and well-ordered perovskite structures observed in the bare 3D and 3D/2D films (Fig. S14).

The XPS findings further prove the differences between 2D capping and amorphous capping. In 4TF-BA-based films, the Pb 4*f* spectrum is broader and has potentially shifted binding energies due to disordered structure compared to the 2D capping layers (Fig. 2D). On the other hand, in 4F-BA-based films, the Pb 4*f* XPS spectra are generally sharper and more distinct, indicating more well-defined bonding and fewer surface defects. The XPS analysis of the Pb 4*f* core levels shows that the full width at half maximum (FWHM) values for both Pb 4*f*_5/2_ and Pb 4*f*_7/2_ peaks are lowest in the 4F-BA-based film (0.972 and 0.863 eV, respectively), indicating a more homogeneous and well-passivated chemical environment. In contrast, the slightly broader peaks in the 4TF-BA-based sample (0.989 and 0.905 eV) suggest increased surface disorder and less effective passivation. These results confirm that the crystalline 2D layer formed by 4F-BA contributes to superior interfacial ordering and defect passivation compared to the amorphous counterpart. Interestingly, the bare 3D perovskite film exhibited intermediate FWHM values (0.985 and 0.883 eV), reflecting moderate surface disorder, which is alleviated by crystalline 2D passivation but exacerbated by amorphous overlayers. Furthermore, the 2D capping layers reduced the Pb^2+^ reduction (metallic Pb) compared to the control and 4TF-BA-based films. XPS analysis of the Pb 4*f*_7/2_ peak revealed a binding energy of 143.58 eV for the bare 3D perovskite film. Upon passivation with an amorphous 2D layer, the peak shifted to 143.12 eV, suggesting increased electron density around Pb^2^⁺ due to disordered local interactions. In contrast, the crystalline 3D/2D heterojunction exhibited a moderate shift to 143.32 eV, consistent with uniform and well-coordinated Pb environments, highlighting the structural and electronic benefits of ordered 2D passivation. Nevertheless, the shift in I 3*d* spectra for 2D’s case was higher than the amorphous one, which makes sense because, in 2D perovskites, iodine tends to be more oxidized or more strongly bonded to Pb^2^⁺, which can lead to higher binding energies for the I 3*d* peaks (Fig. 2E). Further, the less broad and more distinctive peak of C 1*s* in 4F-BA’s case indicates the defined interactions between the spacer cations and the perovskite layer, i.e., a more organized structure as compared to the amorphous one (4TF-BA-based film), as shown in Fig. S16A. The F 1*s* spectra of Fig. S16B confirm the presence of benzamidine on the perovskite film, and the peak belonging to the C-F bond (around 292.39 eV) also disappeared in the case of control films and appeared in 4TF-BA’s case (Fig. 2F, G). This specific peak also discovers another interesting fact, i.e., the reduction in 4F-BA’s case (Fig. 2H). When 4F-BA is incorporated into the 2D perovskite lattice, NH_3_^+^ likely interacts with PbI_6_ octahedral. This interaction can redistribute electron density within the molecule, affecting the C–F bond polarity, as already explained in the charge transfer calculation and formation energies of Fig. 1B, C. If fluorine withdraws less electron density from carbon due to charge delocalization, the binding energy of the C 1*s* (C–F) peak may become weaker in intensity.

PL spectroscopy was used to characterize the perovskite films (without electron extraction layers) to further investigate the charge transfer and recombination behaviors at the perovskite surface. When excitation light was directed from the perovskite side, the PL emission intensity was notably enhanced in the modified films, especially in 4F-BA-treated films (Fig. S17A). The corresponding TRPL measurements were taken to examine carrier dynamics (Fig. S17B). These measurements indicated that 4F-BA passivation was best followed by 4TF-BA and then control. This passivation leads to a reduction in non-radiative recombination with longer PL lifetimes (Table S1), further confirming their role in improving charge carrier retention.

**Fig. 3:**
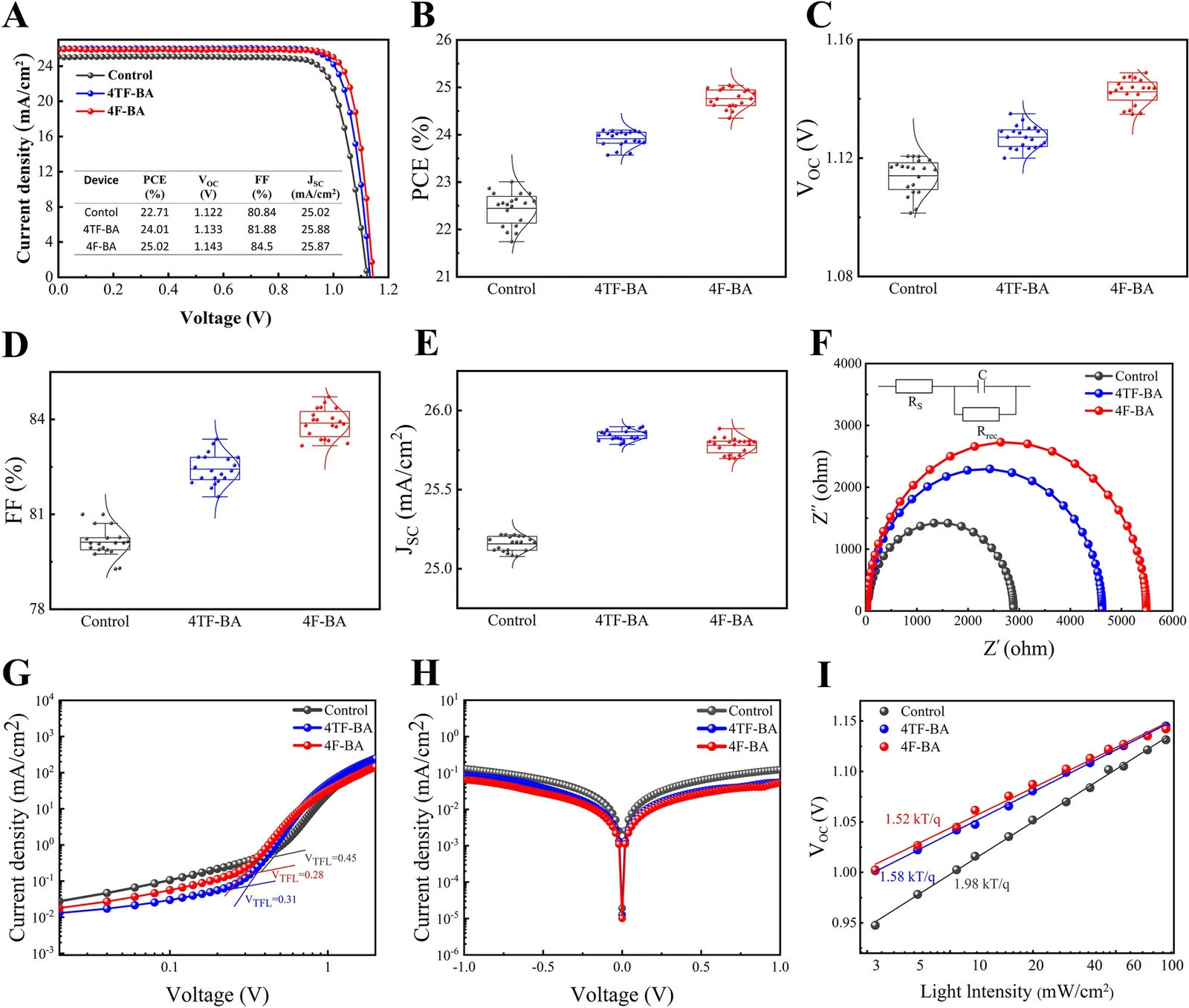
**A** *J–V* curves of PSCs with and without 4TF-BA and 4F-BA treatment. Statistical **B** PCE, **C** *V*_*OC,*_ **D** *FF*, and **E** J_SC_ based on 20 devices from each group. **F** EIS spectra of the control, 4TF-BA, and 4F-BA-based devices, measured under dark conditions at a 0.95 V bias, and the corresponding component values (Table S2) are extracted from the equivalent circuit as shown in the inset (top-right). **G** Space charge-limited current (SCLC) measurements for electron-only devices (FTO/SnO_2_/perovskite/PC_61_BM/BCP/Ag) under dark conditions with and without 4TF-BA and 4F-BA treatment; **H** Dark *J–V* curves and, **I** light intensity dependence *V*_*OC*_ (Suns-*V*_*OC*_) curves of PSCs with and without 4TF-BA and 4F-BA treatment

Building on the advancements observed in the 4TF-BA- and 4F-BA-treated perovskite films, p-i-n PSCs were fabricated, and their *J–V* curves are presented in Figs. 3A and S18. To ensure statistical robustness, 20 devices belonging to each category were fabricated, and their key photovoltaic parameters were analyzed, as shown in Fig. 3B–E. The device treated with 4F-BA achieved a remarkable PCE of 25.02%, accompanied by a current density (*J*_*SC*_) of 25.87 mA cm^−2^, an open-circuit voltage (*V*_*OC*_) of 1.143 V, and an *FF* of 84.5%. The best device based on 4TF-BA achieved an efficiency of 24.01%, with *V*_*OC*_, *J*_*SC*_, and *FF* of 1.113 V, 25.88 mA cm^–2^, and 81.88%, respectively. In contrast, the control device delivered a PCE of 22.71%, with a *J*_*SC*_ of 25.02 mA cm^−2^, *V*_*OC*_ of 1.122 V, and an *FF* of 80.84%. 2D perovskite layer is known to reduce defects and suppress recombination; they help enhance V_OC_ and, potentially, FF. Nevertheless, the amorphous layer is less conductive than the 2D layer; it could increase series resistance, lowering the *FF*. Or in other words, *FF* improved significantly with 2D perovskite passivation, indicating better interfacial quality and reduced recombination, while only moderate enhancement was observed with amorphous passivation, highlighting the importance of an ordered 2D structure for efficient charge extraction. Similar observations have been seen in the EIS results. To further corroborate the *J*_*sc*_ values, EQE measurements were taken (Fig. S19). Integration of the incident photon-to-current conversion efficiency yielded *J*_*SC*_ values of 24.03 mA cm^−2^ for the control device, 24.4 mA cm^−2^ for the 4TF-BA-treated device, and 24.34 mA cm^−2^ for the 4F-BA-treated device, which are consistent with the *J–V* results. The EQE spectrum for the 4F-BA-treated device exhibited a slight enhancement across the visible light range, attributable to improved film quality and more efficient charge transportation. These results highlight the transformative impact of 4F-BA treatment on enhancing device performance and overall efficiency. The same strategy has been applied to the solar cells based on a different absorber layer (Cs_0.05_(FA_0.95_MA_0.05_)_0.95_Pb(I_0.95_Br_0.05_)_3_), which exhibited an enhanced performance when treated with 4F-BA (Fig. S20).

We further evaluated the device through EIS and dark *J–V*. As shown in Fig. 3F, EIS was conducted on devices with different passivation treatments, and Nyquist plots were obtained under dark conditions at a bias voltage of 0.95 V. A single semicircle in the Nyquist plot typically represents the charge recombination resistance (R_rec_) (Table S2). Devices incorporating 4TF-BA and 4F-BA-treated perovskite films exhibited higher R_rec_. Interestingly, lower series resistance (R_s_) is found in the 3D/2D as compared to the 3D/amorphous device, which supports the fact of higher *FF* in the 3D/2D-based device. These results indicate a significant reduction in interface defect-assisted trapping states, which explains the high performance in 4F-BA-treated 3D/2D heterojunction-based devices. The electron trap densities of different perovskite films were investigated using the space charge limited current (SCLC) method on an electron-only device, as shown in Fig. 3G. Compared to the control film, the electron trap density significantly decreased after treatment with 4TF-BA and 4F-BA, from 3.47 × 10^15^ to 1.86 × 10^15^ and 1.02 × 10^15^ cm^−3^, respectively.

Similarly, the dark current was significantly reduced in devices fabricated with 4TF-BA and 4F-BA-treated perovskite films (Fig. 3H) that shows the defect passivation of both types of passivation, i.e., amorphous passivation and 2D passivation. The relationship between the *V*_*OC*_ and light intensity is depicted as suns-*V*_*OC*_ curves in Fig. 3I. The slope of the *V*_*OC*_ versus light intensity curve, given by *k*_*B*_*T/q*, is indicative of trap-assisted recombination, where* k*_*B​*_ is the Boltzmann constant, *T* is the temperature, and *q* is the charge [40]. Compared to the control device, which exhibits a slope of 1.98 *k*_*B*_*T/q*, the devices with 4TF-BA and 4F-BA-treated perovskite layers show slopes of 1.58 and 1.52 *k*_*B*_*T/q*, respectively. The smaller slope values reflect a reduction in non-radiative recombination. These results suggest that the 4TF-BA and 4F-BA treatments effectively mitigate trap-assisted recombination, especially 4F-BA, which further explains the improvements in the 3D/2D-based device's *V*_*OC*_ and *FF*.

It is well known that perovskite distorts into PbI_2_ under high temperatures. We conducted XRD analysis of each perovskite film after prolonged annealing (for 45 min) at 50, 100, and 150 °C (Fig. S21). The control device exhibited an enhanced PbI_2_ peak at 100 °C, while the 4TF-BA-based film exhibited a prominent peak at 150 °C. 4F-BA outperformed the control and 4F-BA-based films and shows a minute peak of PbI_2_ even when annealed at 150 °C. This also suggests the heat stability of crystalline 2D-based devices as compared to passivated and control perovskite. It is evident that the PbI_2_ converts to metallic Pb (Pb^0^) under light, especially UV light, which further retards the stability of devices [41, 42]. The suppression in the Pb^0^ was noticed in 4F-BA-based perovskite film when the XPS Pb 4f of devices was measured after UV light soaking for seven days (Fig. S22A). Accordingly, after 400 h of continuous illumination, the devices retained 88.1%, 84.8%, and 80.3% of their initial power conversion efficiencies for the 4F-BA, 4TF-BA, and control samples, respectively (Fig. S22B).

To assess the long-term stability, unencapsulated perovskite solar cells were subjected to natural aging under various environmental conditions: at 25 °C in a nitrogen atmosphere, at 65 °C in a nitrogen atmosphere, and at 25 °C with 30%–40% relative humidity (Fig. S23). After 1200 h of exposure, the control device retained 76.2%, 47.3%, and 65% of its initial efficiency under nitrogen, high temperature (at 65 °C), and humidity conditions (RH 30%–40%), respectively. In contrast, the 4TF-BA-treated device preserved 89.6%, 74.2%, and 80.6% of its initial efficiency under the same conditions. The 4F-BA-treated 3D/2D film-based device exhibited the highest performance by maintaining 93.4%, 80.3%, and 90.2% of its initial efficiency under these respective conditions. Additionally, water contact angle measurements were taken to evaluate the surface hydrophobicity. Compared to the untreated perovskite, the contact angles increased from 43.7° to 49.6° and 53.2° for the 4TF-BA and 4F-BA-treated perovskites, respectively, as shown in Fig. S24. As the fluorinated devices are hydrophobic, the devices based on 4TF-BA exhibited the highest hydrophobicity, and as usual, the 2D perovskites are more hydrophobic than the other types of passivation layers [20]. We further conducted the prolonged water droplet test for 4 h in open air. A comparison of the perovskite films (fresh, after 2 h, and after 4 h) is shown in Fig. S25. In the area of the droplet, perovskite turns yellow after 2 h in each case, while it completely vanishes in the case of the control and 4TF-BA case. However, the 4F-BA-based film stays yellow even after 4 h. This unique prolonged water droplet test also suggests the high hydrophobicity of the 3D/2D film. These findings indicate that the 4F-BA-treated perovskite film exhibits the highest resistance to moisture, which is consistent with the observed stability results.

## Conclusions

In this novel study, we explored how a minute change from a similar molecule can influence the electronic properties of the organic cation that further influences the formation of 2D perovskite structures on 3D absorber films, as demonstrated by XRD, SEM, AFM, and GIWAXS measurements. These experimental findings are supported by our theoretical calculations, further showing that the distinction between incomplete amorphous perovskites and well-defined 2D perovskites at the surface of control films is attributed to the intramolecular charge distribution. This distribution subsequently impacts the hydrogen bonding between the NH_3_^+^ group and the iodine in the octahedral PbI_6_ structure. While the amorphous films exhibited defect passivation capabilities and the devices made from these films showed better performance than the control devices, the 3D/2D films excelled in both device performance and long-term stability.

## Supplementary Information

Below is the link to the electronic supplementary material.

Supplementary file1 (DOCX 6365 kb)
