# Supplementary material for: Differentiating the 2D Passivation from Amorphous Passivation in Perovskite Solar Cells
Source: Nanomicro Lett. 2025 Sep 8;18:62. doi: 10.1007/s40820-025-01913-y (PMC12420534; doi:10.1007/s40820-025-01913-y)
Supplement: Supplementary file 1 — Supplementary file1 (DOCX 6365 kb) [file 40820_2025_1913_MOESM1_ESM.docx]

**Supporting Information**

**Differentiating the 2D Passivation from Amorphous Passivation in perovskite solar cells**

Xiaojian Zheng^1,2,#^, Shehzad Ahmed^3,#^, Yu Zhang^1,#^, Guoqiang Xu^1,2^, Junyu Wang^1^, Di Lu^1^, Tingshu Shi^1^, Jun Tang^1^, Lei Yan^2^, Wei Chen^4^, Peigang Han^1^, Zhixin Liu^1,*^, Danish Khan^1,^*, Xingzhu Wang ^2,^ *, Zeguo Tang^1 ,^ *

^1^ College of New Materials and New Energies, Shenzhen Technology University，Lantian Road 3002, Pingshan, Shenzhen 518118, China

^2^ Engineering and Research Center for Integrated New Energy Photovoltaics & Energy Storage Systems of Hunan Province and School of Electrical Engineering, University of South China, Hengyang 421001, Hunan, China

^3^ China-UK Low Carbon College, Shanghai Jiao Tong University, Lingang, Shanghai 201306, China

^4^ College of Engineering Physics, Shenzhen Technology University, Shenzhen, Guangdong 518118, China

*Corresponding author. E-mail: liuzx@sustech.edu.cn, khandanish@sztu.edu.cn, wangxz@sustech.edu.cn, tangzeguo@sztu.edu.cn

^#^These authors contributed equally to this work.

**Computational Details**

All first-principles computations have been performed utilizing the Vienna Ab initio Simulation Package (VASP) grounded in density functional theory (DFT) [1]. The exchange-correlation interactions were characterized using the generalized gradient approximation (GGA) based on the Perdew-Burke-Ernzerh of (PBE) functional [2]. The projector-augmented wave (PAW) approach was utilized to characterize the interaction between core and valence electrons [3]. The PbI_6_ monolayer was represented as a periodic two-dimensional slab with an adequately large vacuum zone (~20 Å) in the out-of-plane direction to prevent interactions between periodic imagery. The plane-wave basis set was augmented with an energy cutoff of 550 eV to guarantee convergence. The Brillouin zone was sampled with a Γ-centered Monkhorst-Pack k-point mesh of 3×3×1 for structural relaxations and 5×5×1 for electronic structure computations [3]. Structural optimizations were conducted until the residual forces on each atom fell below 0.001 eV/Å. To examine the adsorption characteristics of the 4TF-BA and 4F-BA molecules on the PbI_6_ monolayer, we calculated the formation energy (*E _form_*) utilizing the subsequent equation:

*E _form_ = E _adsorbed system_ - (E_PbI6_ + E _molecule_)*

where *E _adsorbed system_* denotes the total energy of the PbI_6_ monolayer with the adsorbed molecule, *E_PbI6_* represents the energy of the pristine PbI_6_ monolayer, and *E _molecule_* signifies the energy of the isolated molecule [4]. Charge transfer study was conducted utilizing Bader charge calculations and charge density differential plots to illustrate charge redistribution during adsorption [5]. The charge transfer density (*Δρ*) was calculated as follows:

*Δρ* = *ρ _adsorbed system_* - *(ρ_PbI6_* + *ρ _molecule_)*

where *Δρ* denotes the charge density of the corresponding components. To further explore the electronic interactions at the molecule-substrate interface, the crystal orbital Hamilton population (-COHP) and projected crystal orbital Hamilton population (-pCOHP) analyses were conducted. These calculations provided insights into the bonding and antibonding interactions between the molecular orbitals and the PbI_6_ substrate. The LOBSTER code was implemented within VASP to compute the -COHP and -pCOHP, enabling a detailed analysis of orbital interactions and bonding characteristics [6]. All calculations were performed with van der Waals (vdW) interactions using DFT-D3 correction scheme to accurately describe the adsorption characteristics of 4TF-BA and 4F-BA on the PbI_6_ layer.

**The carrier lifetime calculation:**

We studied the charge recombination dynamics and used the following formula to fit the TRPL decay curve:

y $=$ y_0_ $+$ A_1_exp$\left( \frac{-t}{\tau_{1}} \right)$ $+$ A_2_exp$\left( \frac{-t}{\tau_{2}} \right)$

(1)

where *A*_1_ and *A*_2_ are relative amplitudes, and *τ*_1_ and *τ*_2_ represent the lifetimes for fast and slow recombination, respectively. The average lifetime of the nonradiative recombination process can be calculated according to the following formula:

(2)

$\tau$_ave_ $=$ $\frac{\sum A_{i}\tau_{i}^{2}}{\sum A_{i}\tau_{i}}$

**Electrochemical impedance spectroscopy (EIS) and capacitance-voltage spectrum (C-V):**

Capacitance- voltage spectrum (C-V) has been studied by electrochemical workstation. The depletion layer capacitance can be approximated as a function of the applied bias-voltage:

C $=\sqrt{\frac{q\varepsilon_{r}N}{2(V_{bi}-V)}}$

(3)

where *q* is the element charge, *ε*_r_ is the relative dielectric constant, *N* is the charge carrier density, and *V*_bi_ is the built-in potential.

**Space charge limited current (SCLC):**

The PCE and SCLC of perovskite solar cells were calculated by recording the current density-voltage (*J-V*) curves on a solar simulator connected to a Keithley 2400 Digital Source Meter under AM1.5G irradiation in the dark. The light intensity was calibrated with a standard silicon photodiode. The scan rate was fixed at 20 mV s^-1^ while the delay time was set to 0.01 s. A metal mask defined the active area with a size of 0.06 cm^2^. The trap density (*N*_trap_) is calculated by equation:

N_trap_ $=$ $\frac{{2C}_{g}V_{TFL}}{qAL}$

(4)

where *C*_g_ is the geometrical capacitance of the perovskite layer, which is obtained by the C-V spectra of the corresponding devices, *V*_TFL_ is the trap-filled limit voltage obtained through the dark *J-V* curve, *q* is the elementary charge, *A* is the active area, and *L* is the thickness of perovskite film.

**Supporting Figures**


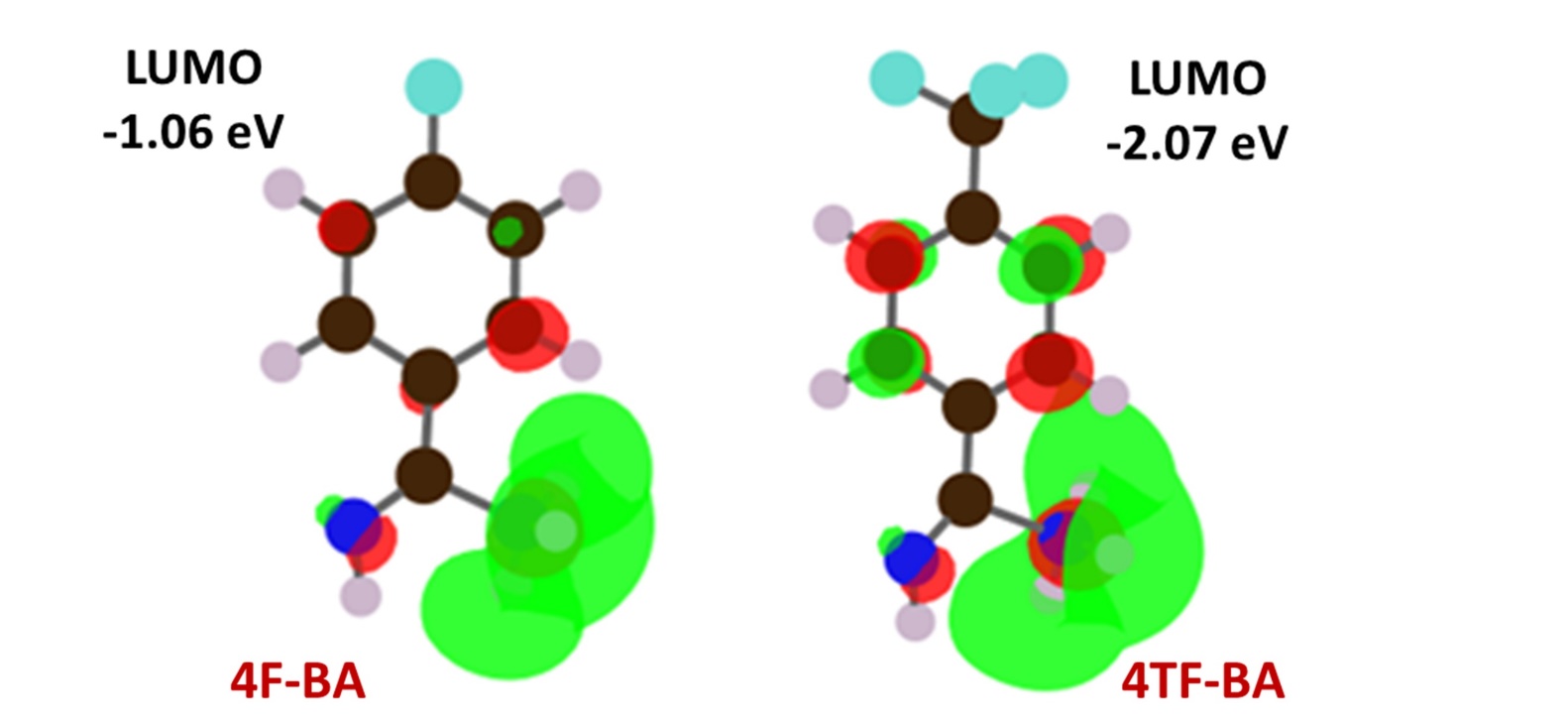


**Fig. S1**. LUMO isosurfaces and levels of 4F-BA and 4TF-BA.


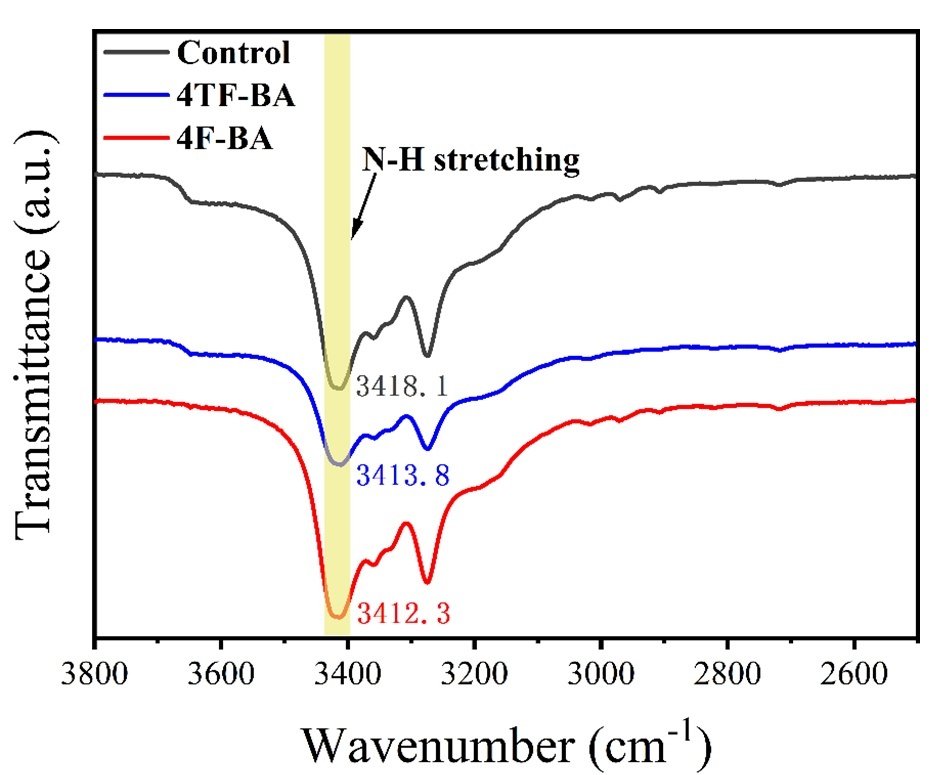


**Fig. S2**. The FTIR spectra of control, 4TF-BA, and 4F-BA-based films.

**
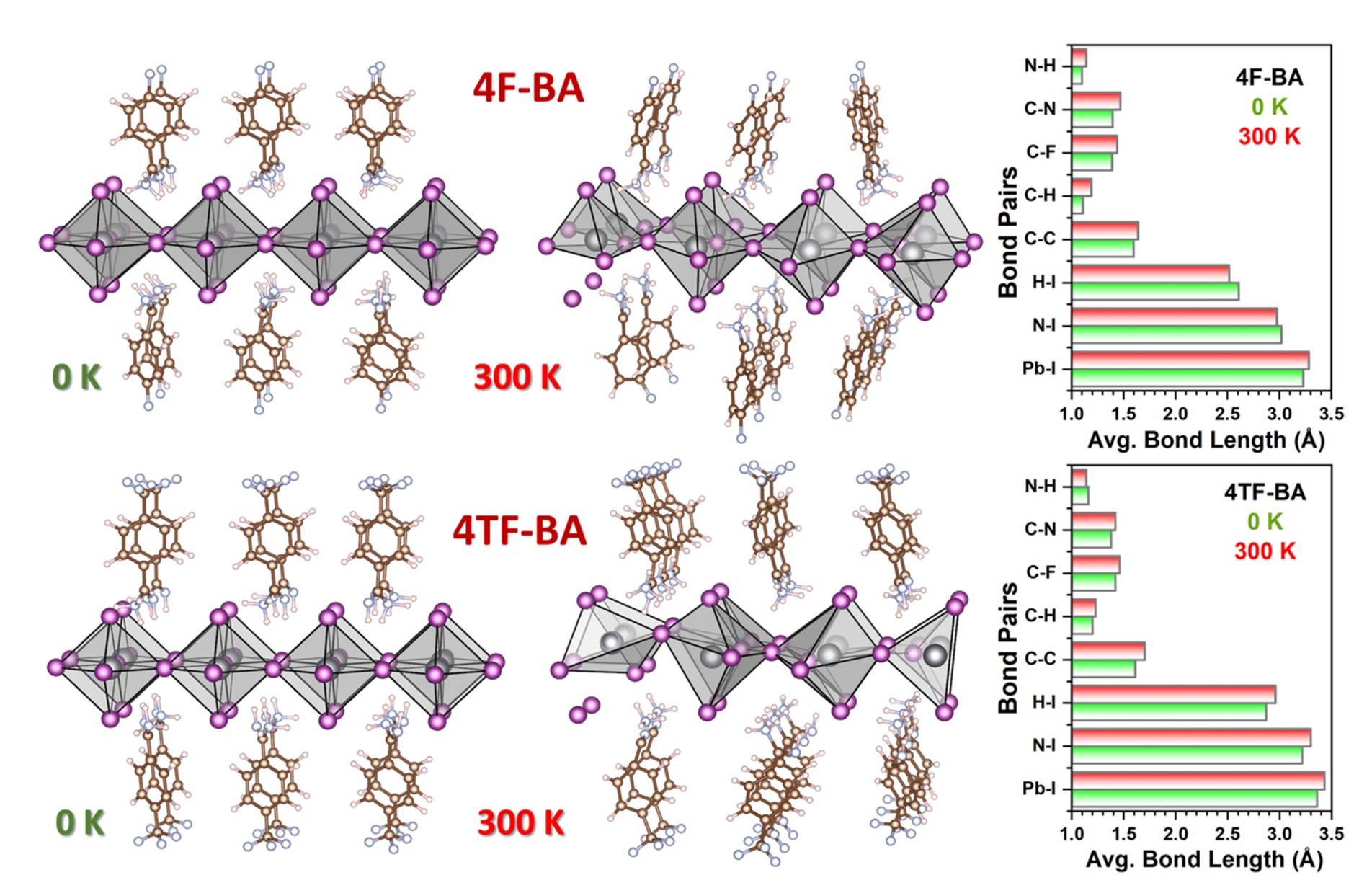
**

**Fig. S3**. (a) Structural snapshots before and after AIMD simulations at 300 K, illustrating better thermal stability in 4F-BA. And, the bond length comparison before and after AIMD. 4F-BA exhibits shorter N–I and H–I distances at 300 K, indicating stronger interfacial binding than 4TF-BA.

**
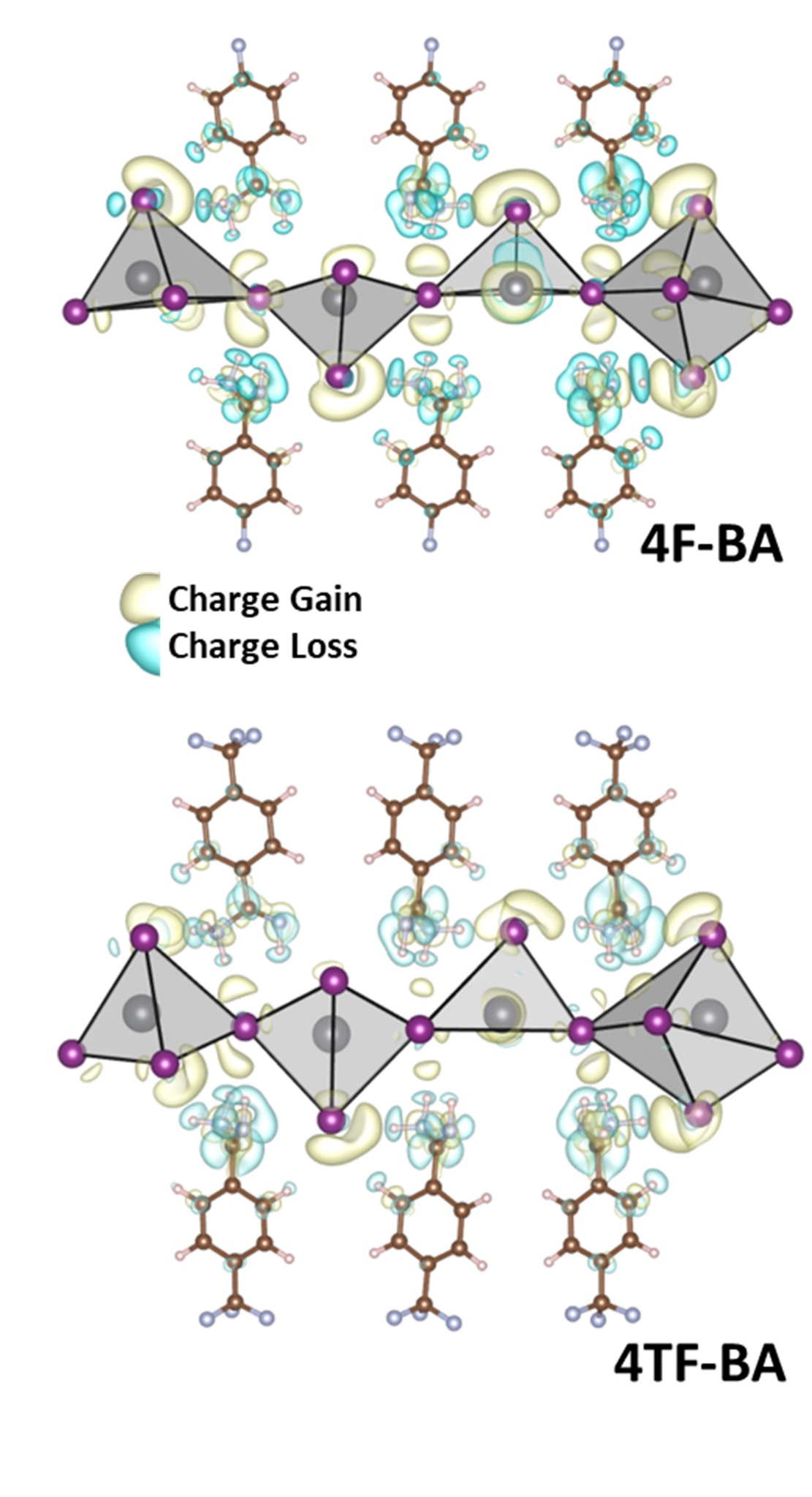
**

**Fig. S4.** Charge density difference plots showing greater interfacial charge transfer for 4F-BA than 4TF-BA.

**
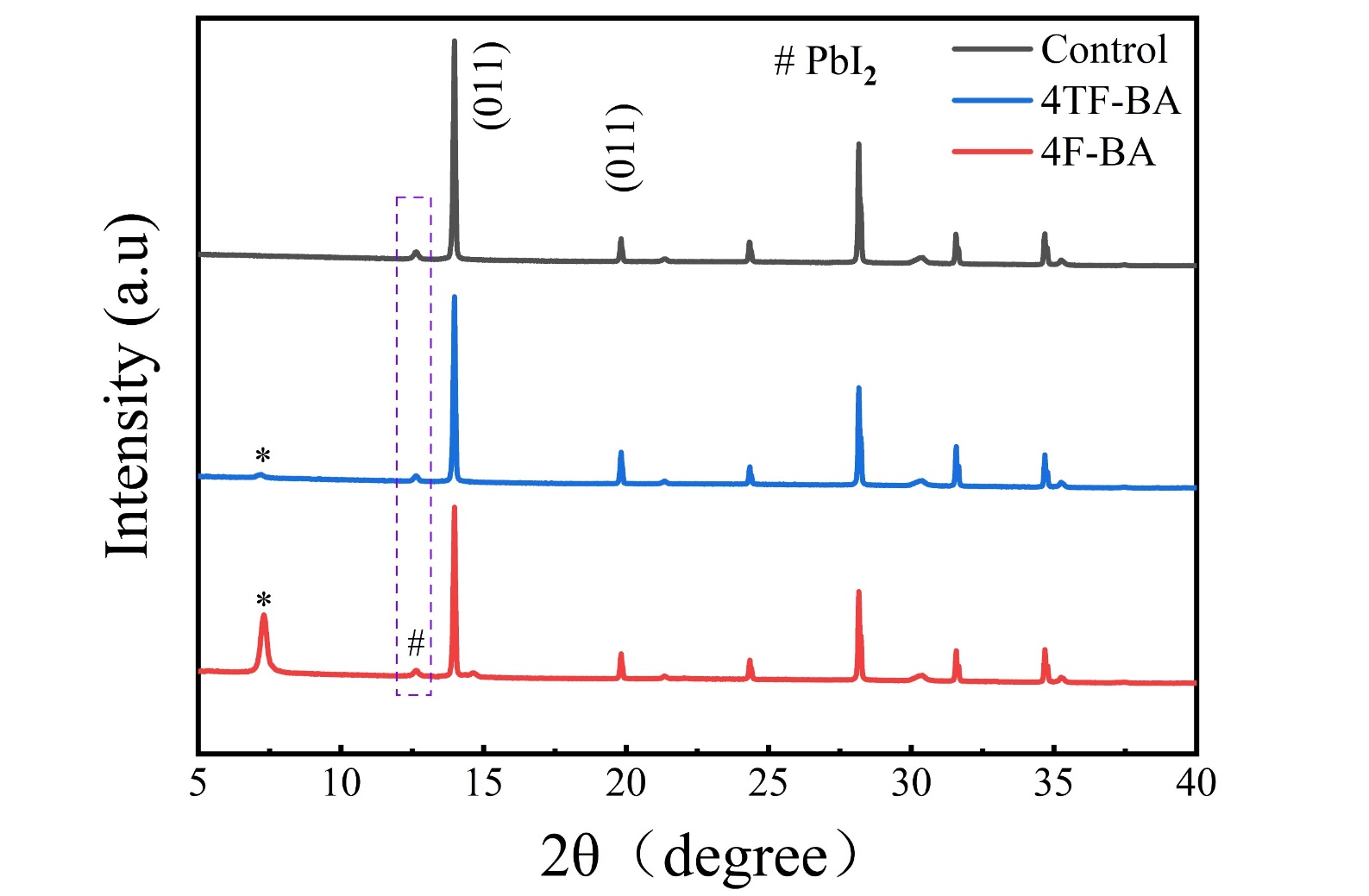
**

**Fig. S5**. XRD results of control and modified films via 4TF-BA and 4F-BA.


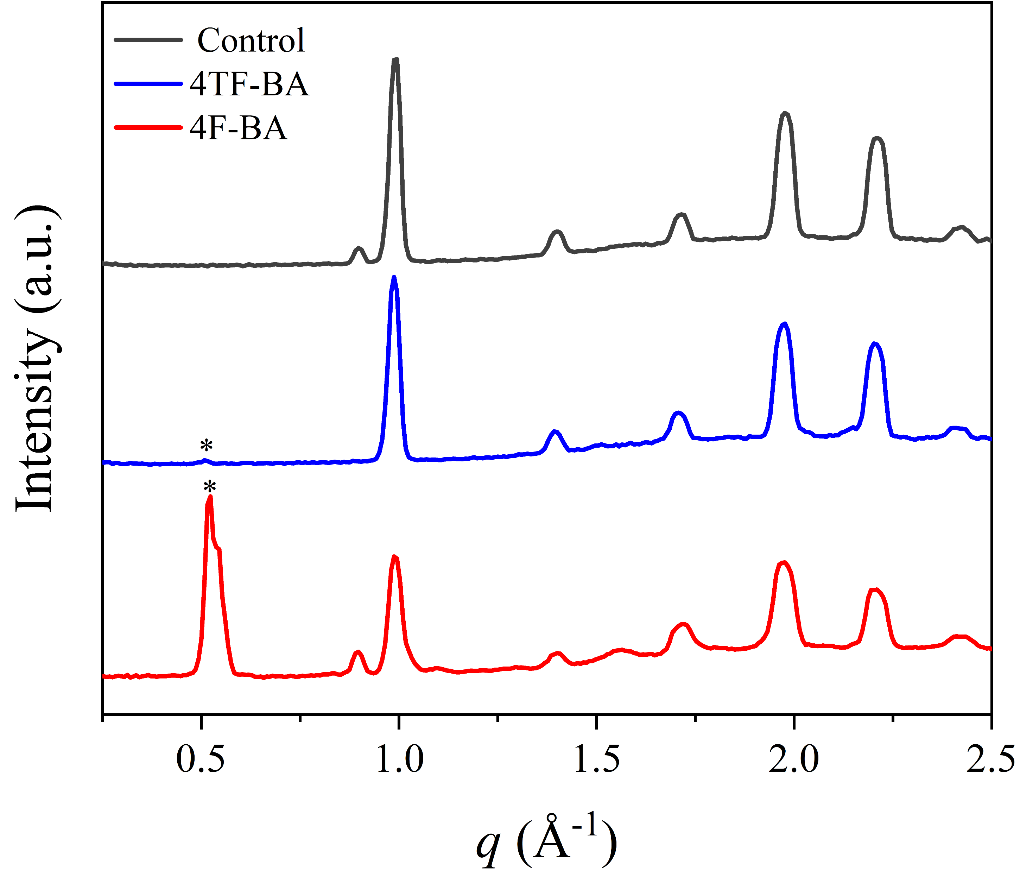


**Fig. S6.** Intensity profiles along the qz axis of the corresponding perovskite film extracted from GIWAXS data for each case.


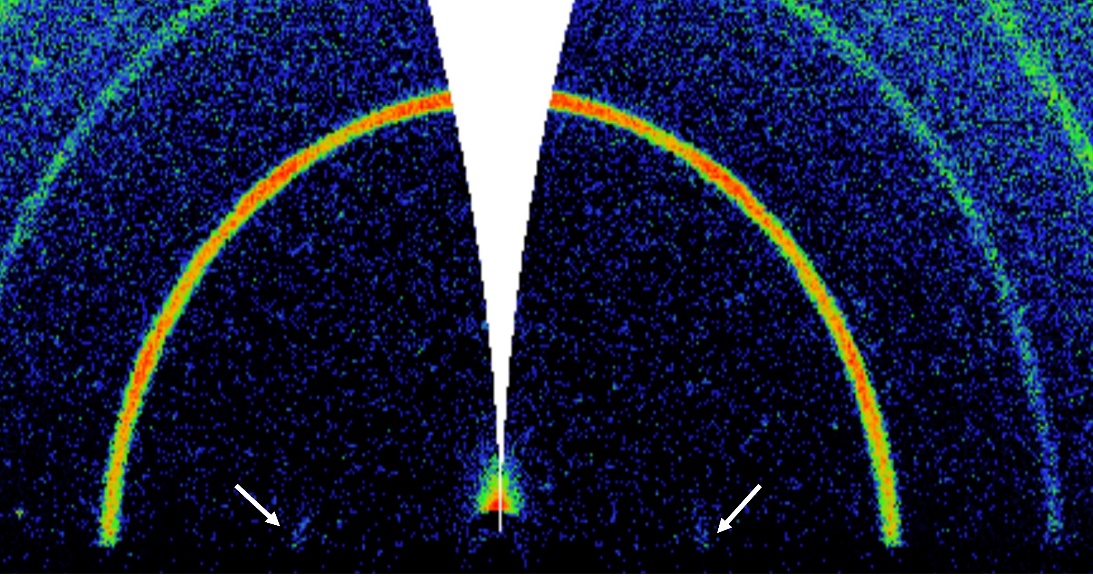


**Fig. S7**. Zoomed-in version of GIWAXS of 4TF-BA-based perovskite films. Very faint signal as highlighted by white arrows.


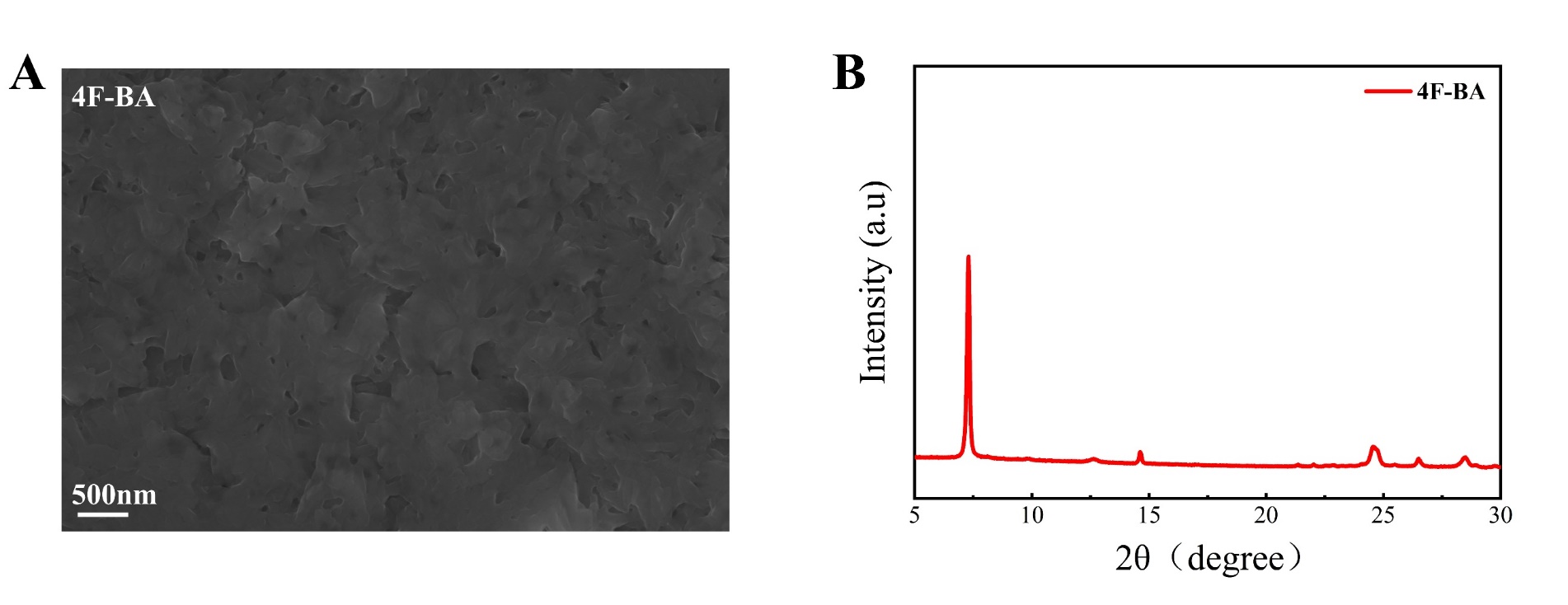


**Fig. S8**. (A) SEM image and (B) XRD graph of pure 2D Ruddlesden–Popper perovskite film (4F-BA)₂Pb(ICl)₄.


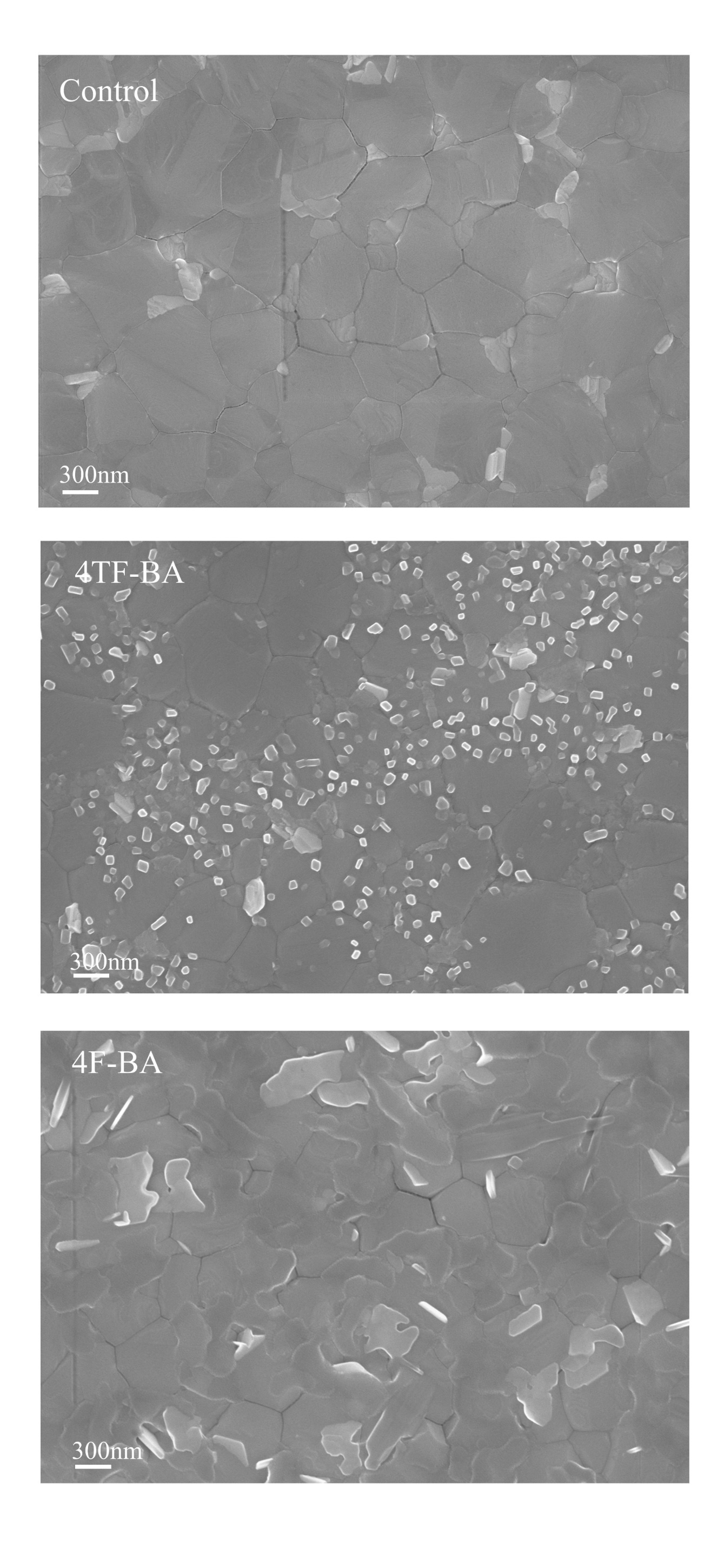


**Fig. S9.** Top SEM images of the control perovskite and perovskite treated with 4TF-BA and 4F-BA.

**
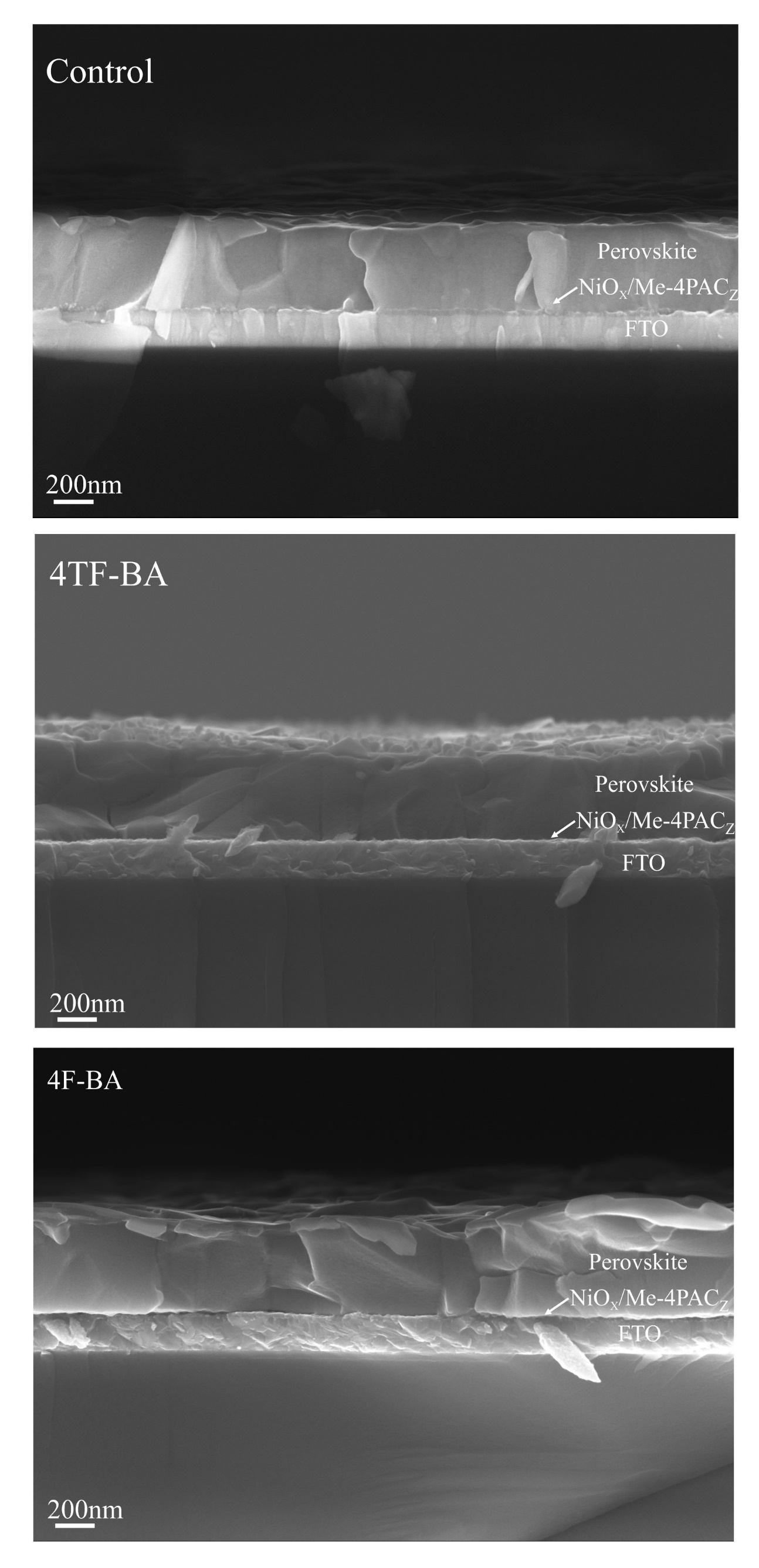
**

**Fig. S10.** Cross-sectional SEM images of the control perovskite film and the films treated with 4TF-BA and 4F-BA.

**
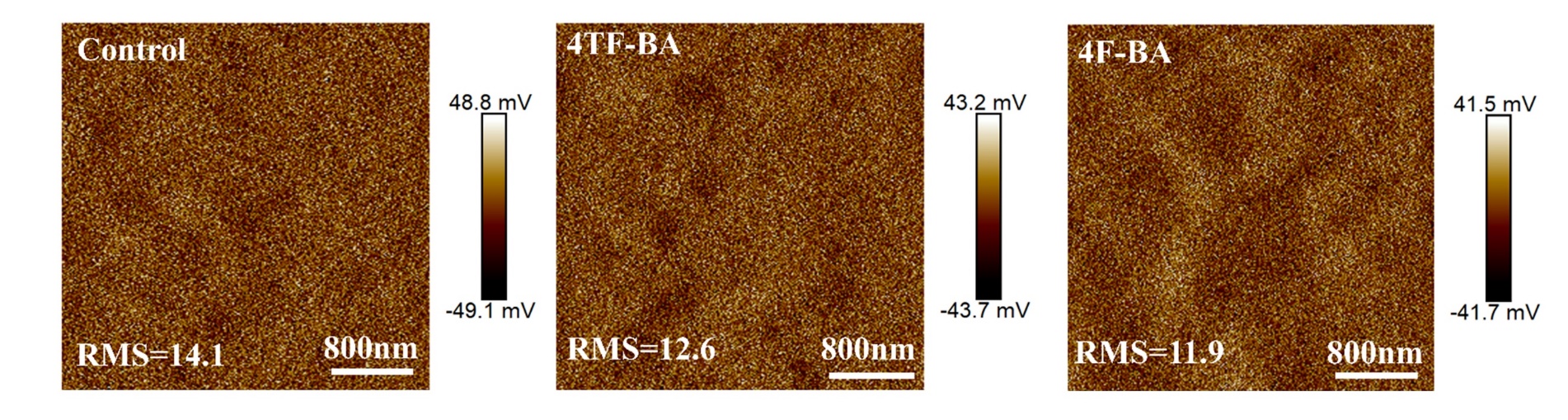
**

**Fig. S11.** Surface contact potential difference distribution images, measured by KPFM of perovskite films without or with 4TF-BA and 4F-BA treatments.


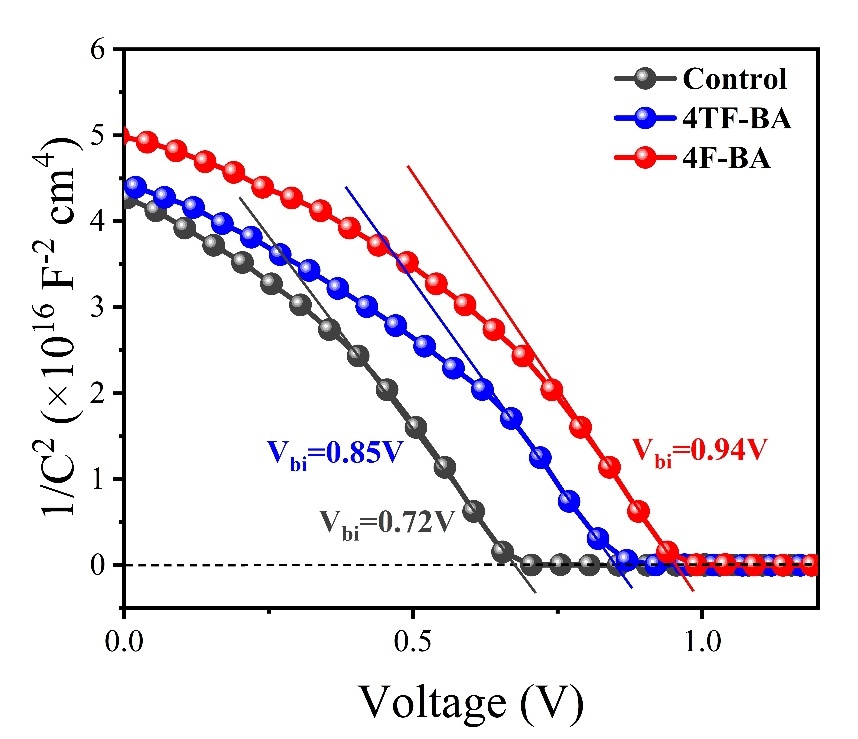


**Fig. S12**. Mott-Schottky plots of control and modified devices.

**
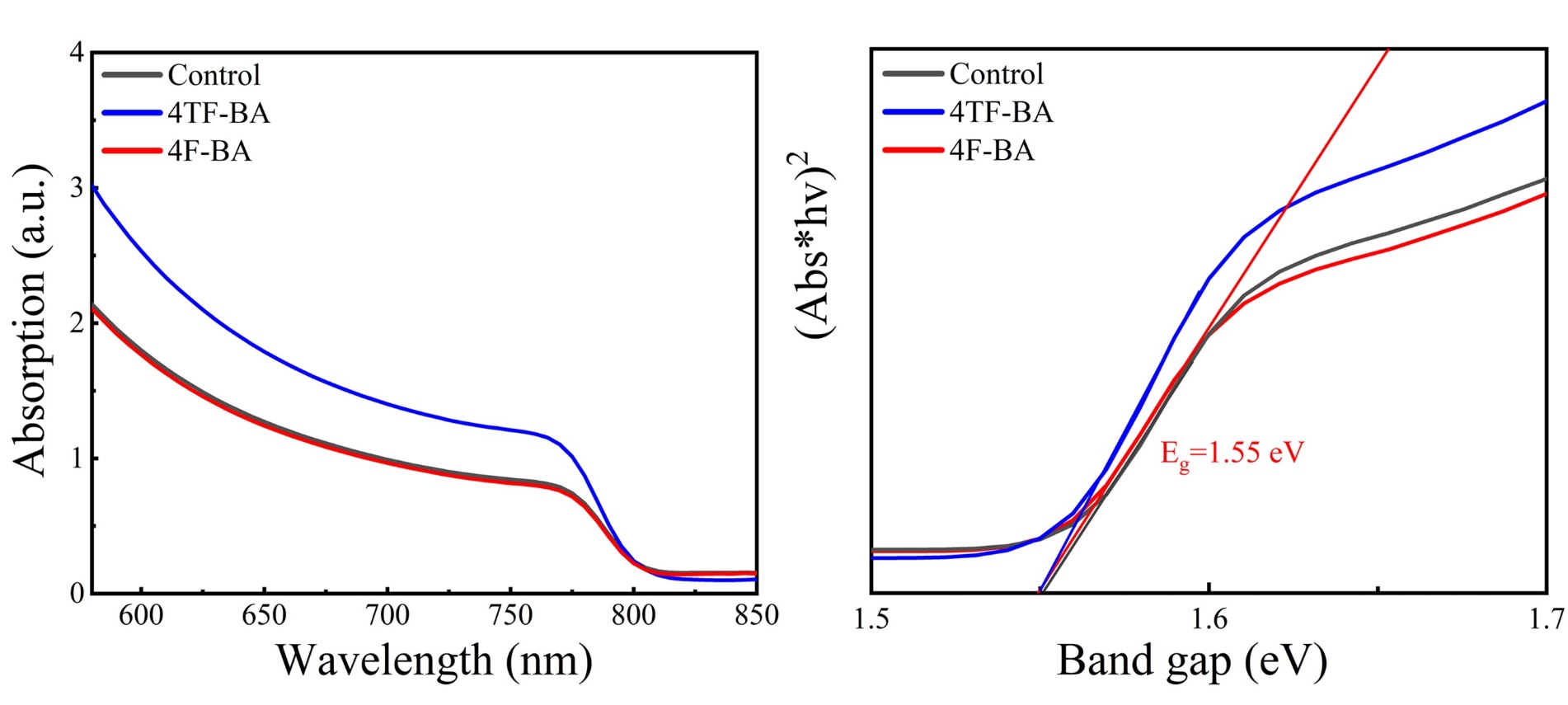
**

**Fig. S13.** UV-vis absorption spectra of control and modified films (left panel) and the extracted Tauc plots showing the band gap of 1.55 eV in each case.


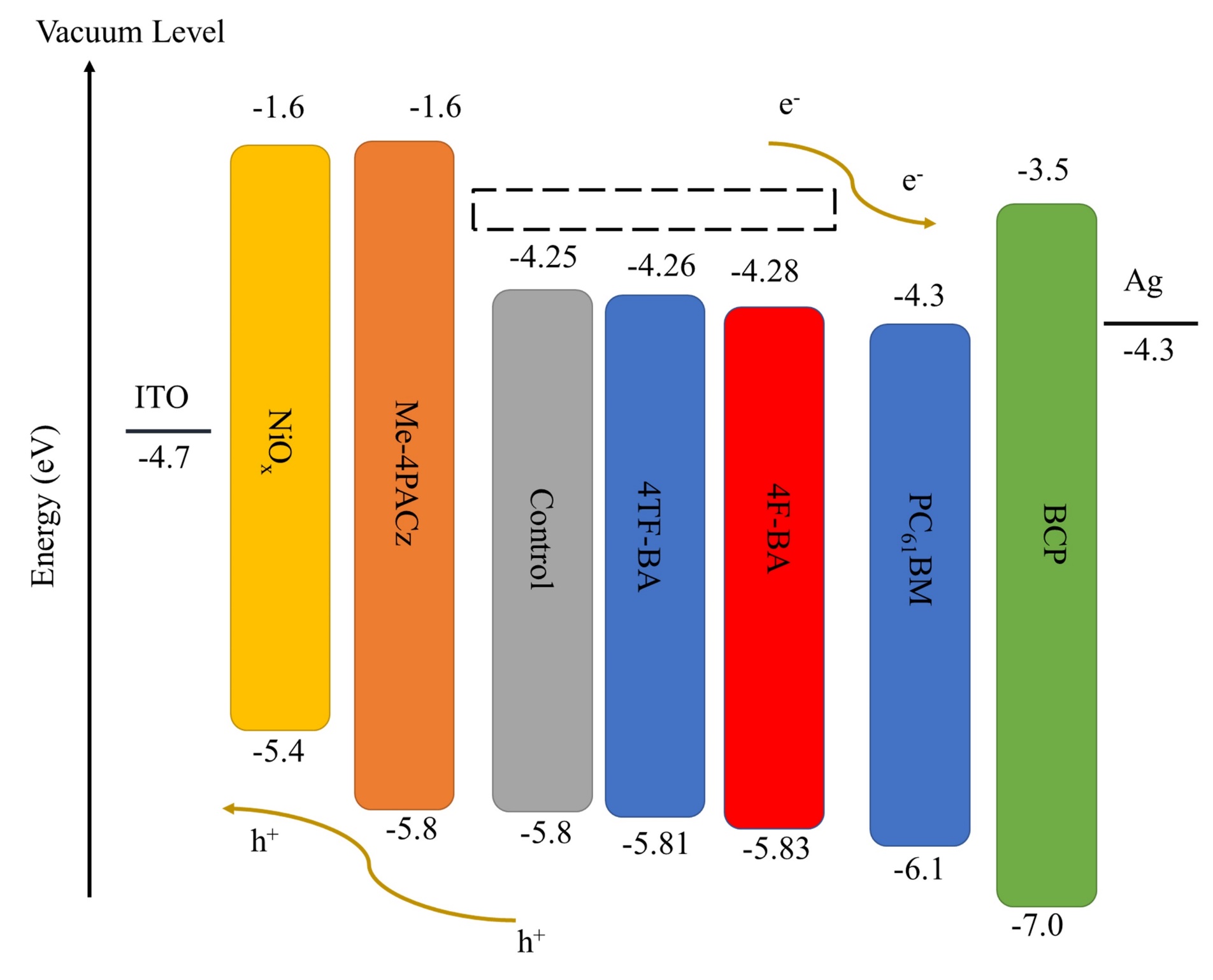


**Fig. S14.** The energy level diagram of the layers of our as-fabricated p-i-n device.


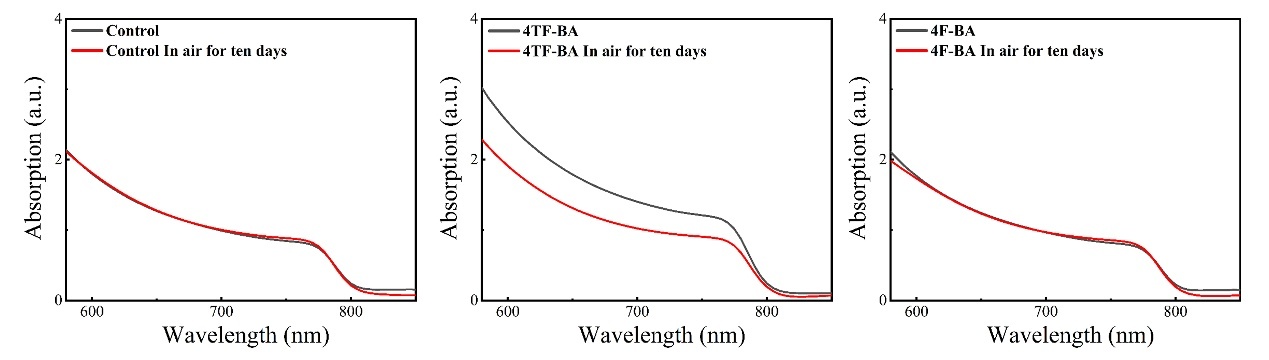


Fig. S15. Absorption comparison before and after aging in ambient air for 10 days, obtained from the UV-vis test.


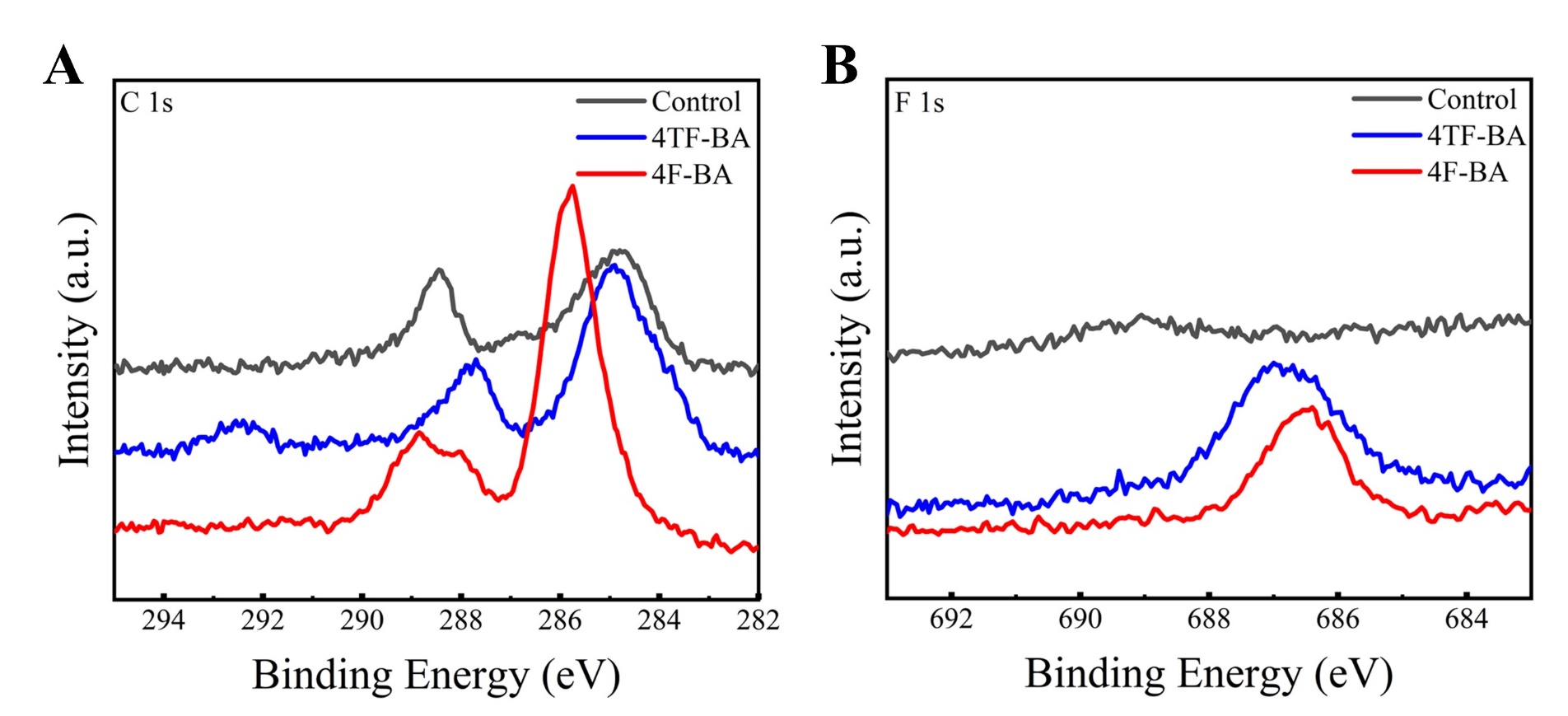


**Fig. S16.** XPS spectra of (A) C 1s and (B) F1s core levels of control,4TF-BA and 4F-BA treated perovskite films.


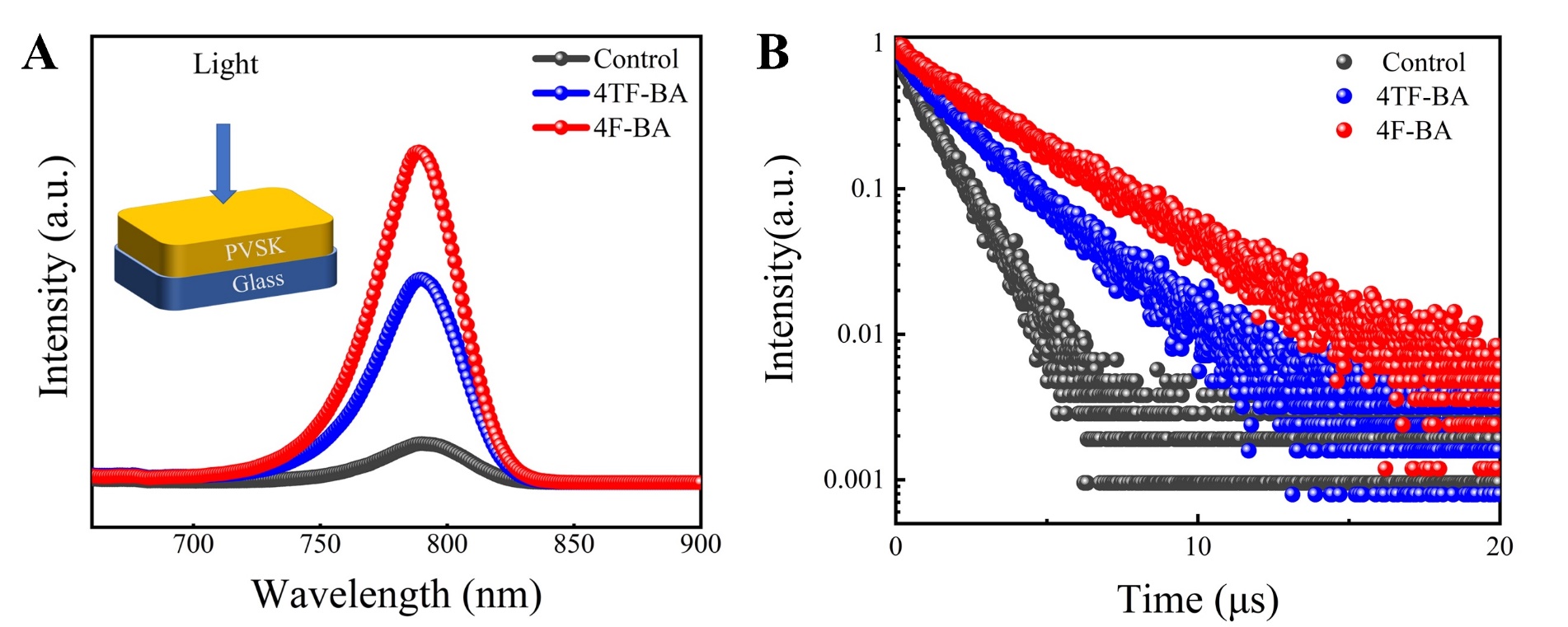


**Fig. S17.** (A) PL intensity and (B) the corresponding TRPL of the control, 4TF-BA, and 4F.

**
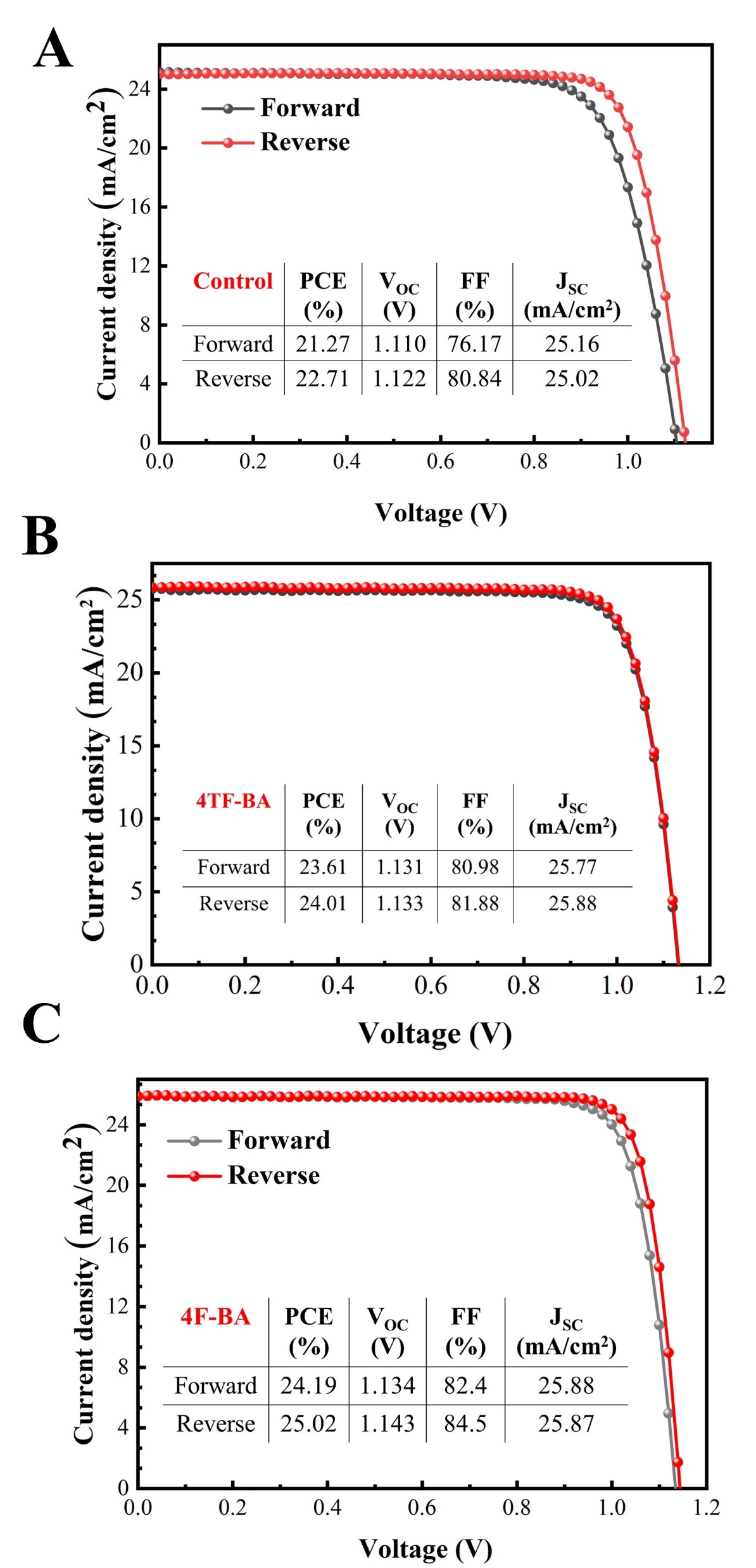
**

**Fig. S18.** *J-V* curves of the best-performing (A) control device, (B) 4TF-BA-based device, and (C) 4F-BA-based device.

**
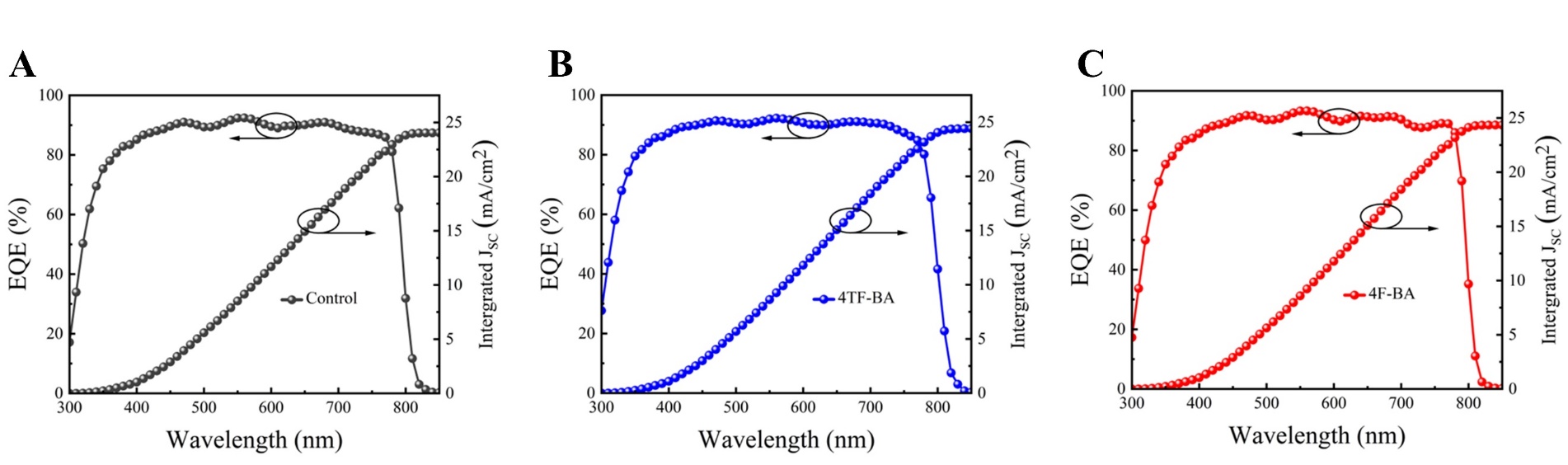
**

**Fig. S19.** External quantum efficiency curves and integrated *J_SC_* of (A) control, (B) 4TF-BA, and (C) 4F-BA-based device.


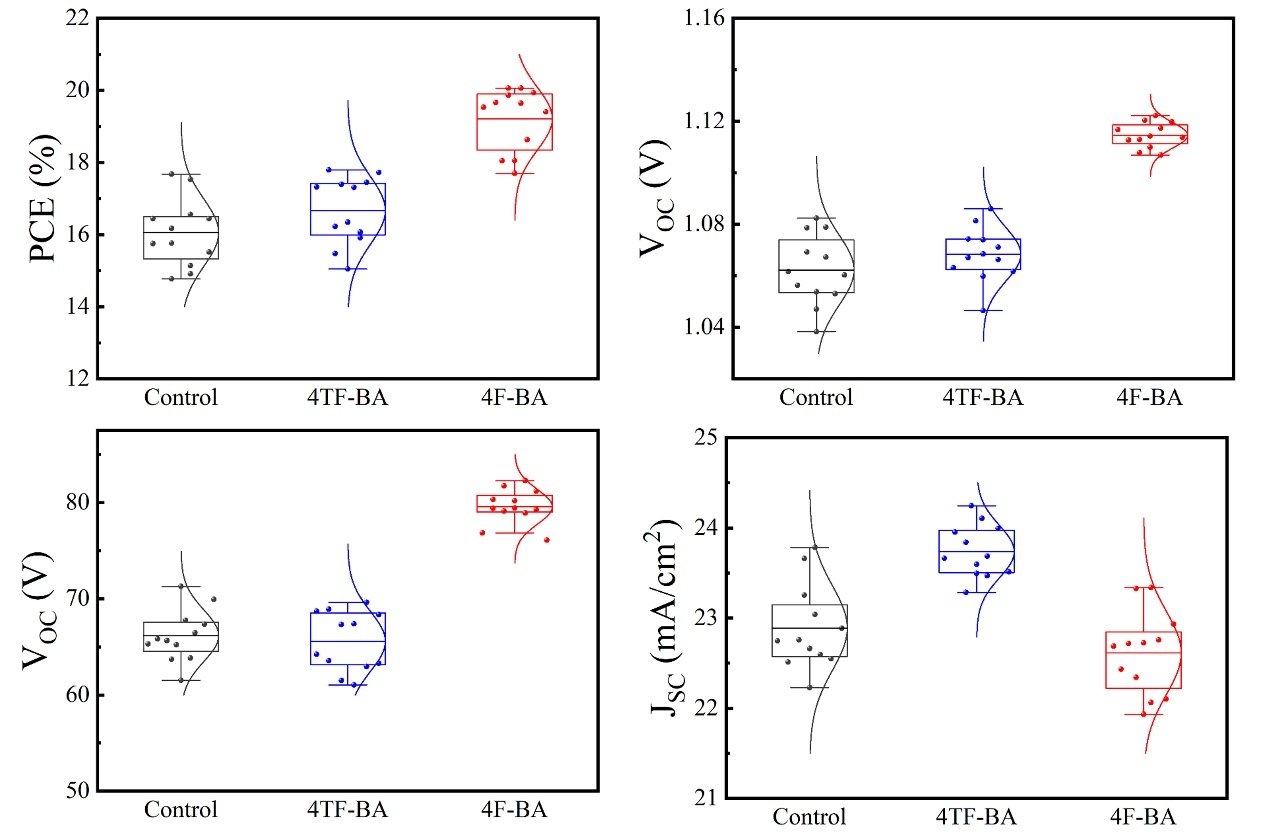


**Fig. S20**. Photovoltaic performances of the devices based on Cs_0.05_(FA_0.95_MA_0.05_)_0.95_Pb(I_0.95_Br_0.05_)_3_ perovskite in each case.


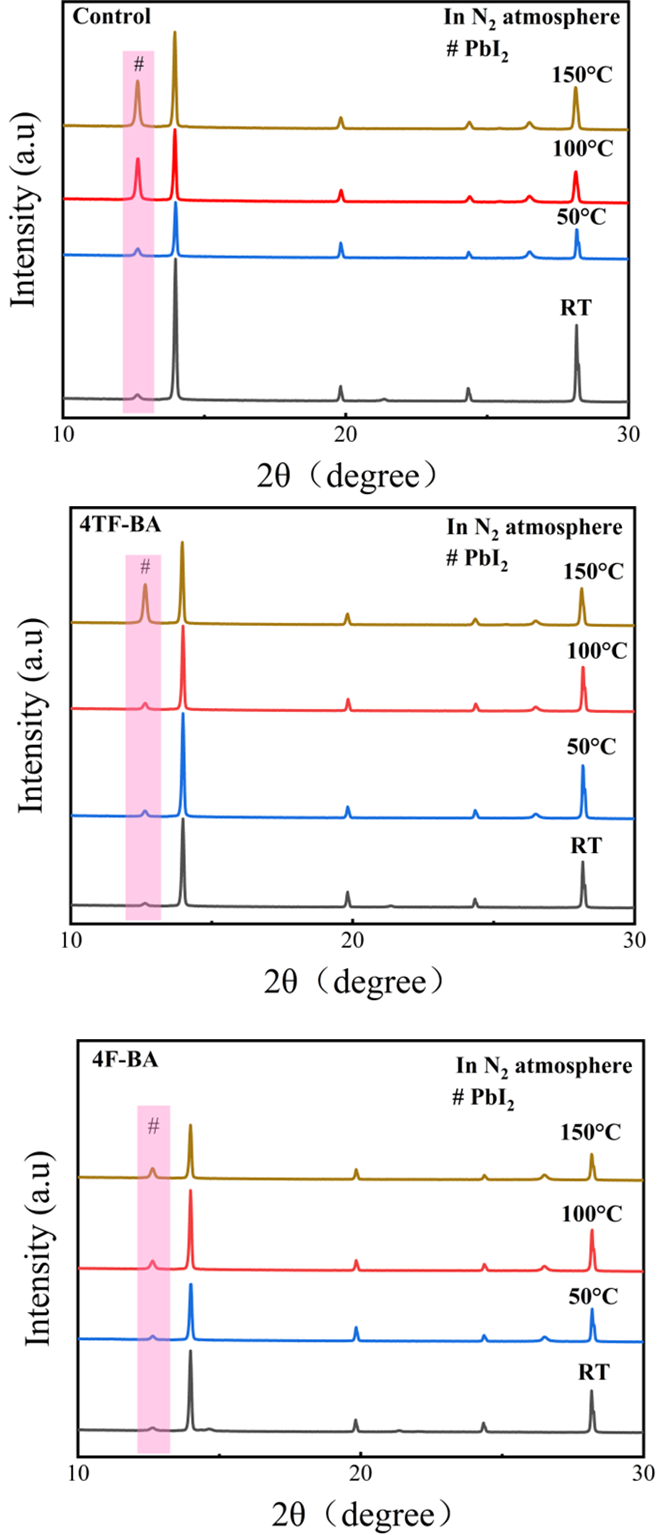


**Fig. S21**. XRD graphs under different annealing temperature of control, 4TF-BA-based, and 4F-BA-based perovskite films.


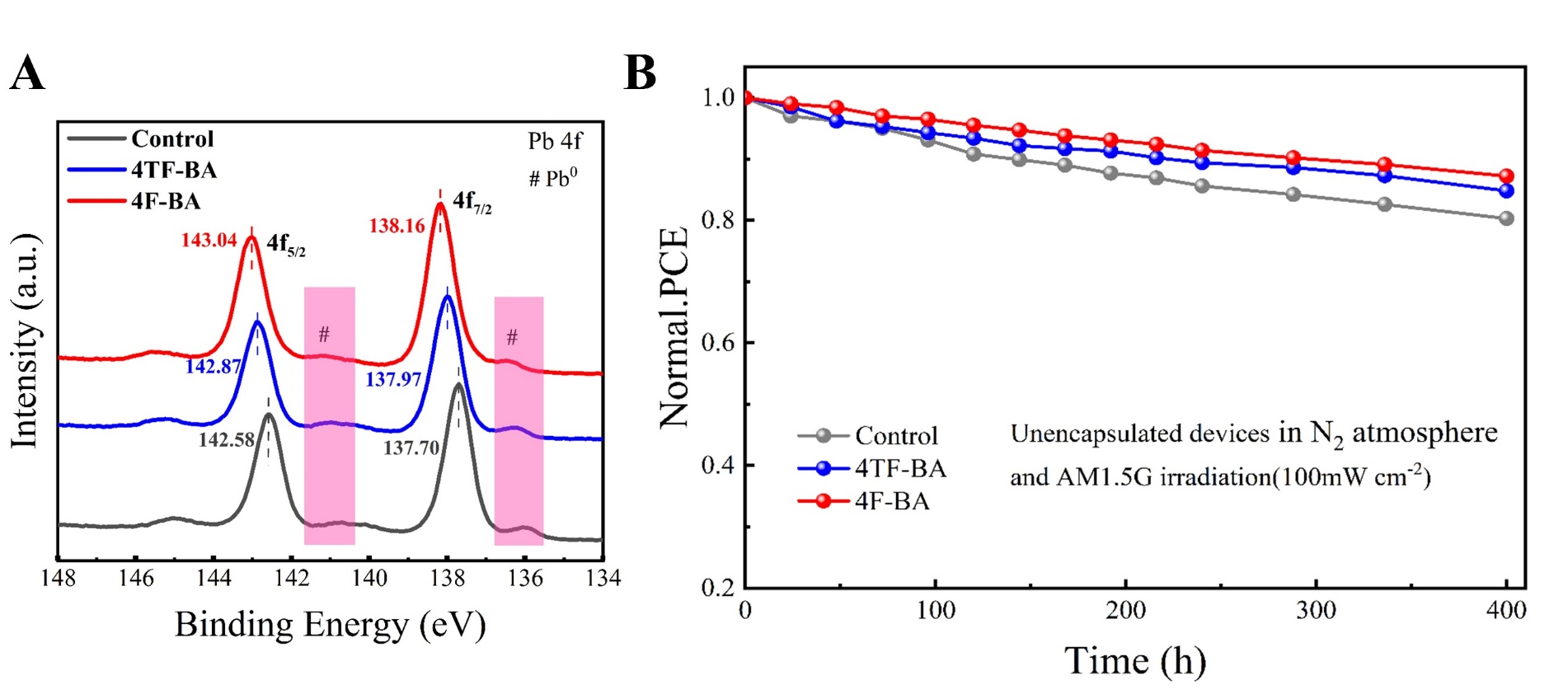


Fig. S22. (A) Pb 4f XPS comparison of the control and modified films after UV light soaking for 7 days. (B) Long-term stability tracking under continuous illumination for 400 hours in an N2 environment.


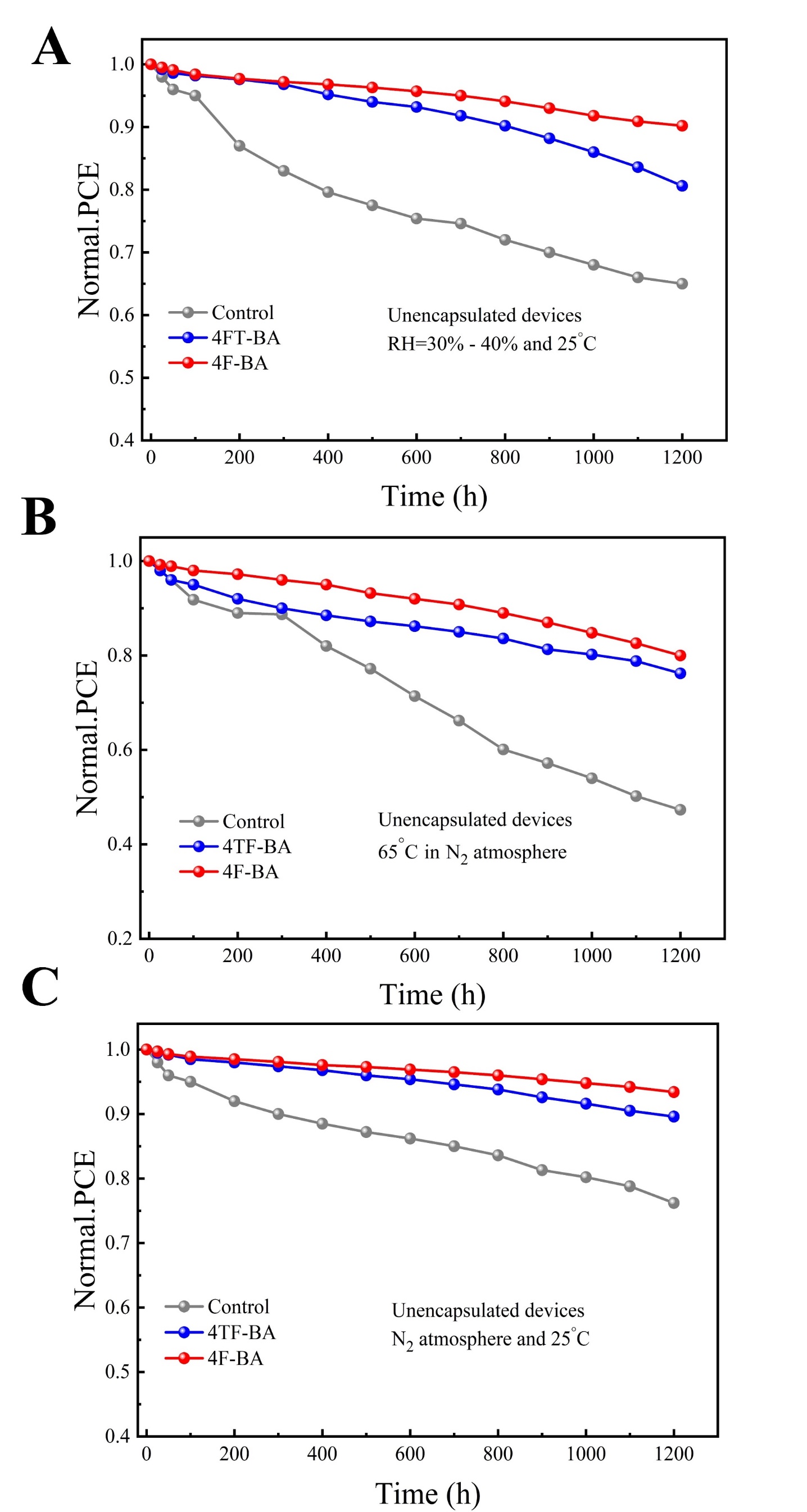


**Fig. S23.** Long-term stability of unencapsulated devices (A) stored in an N_2_ atmosphere at 25°C, (B) stored in an N_2_ atmosphere at 65°C, and (C) stored under ambient conditions (25°C, 30–40% relative humidity).


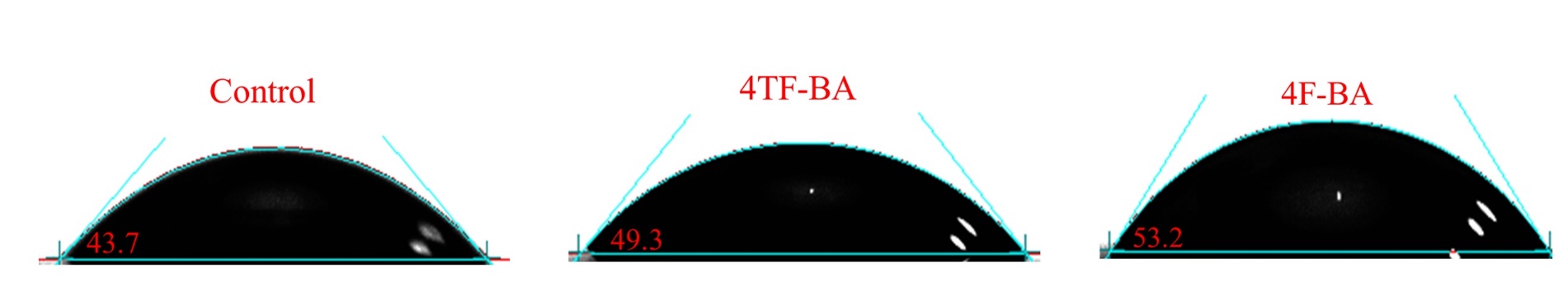


**Fig. S24**. The water contact angle measurements of the Control, 4TF-BA, and 4F-BA-treated perovskite films.


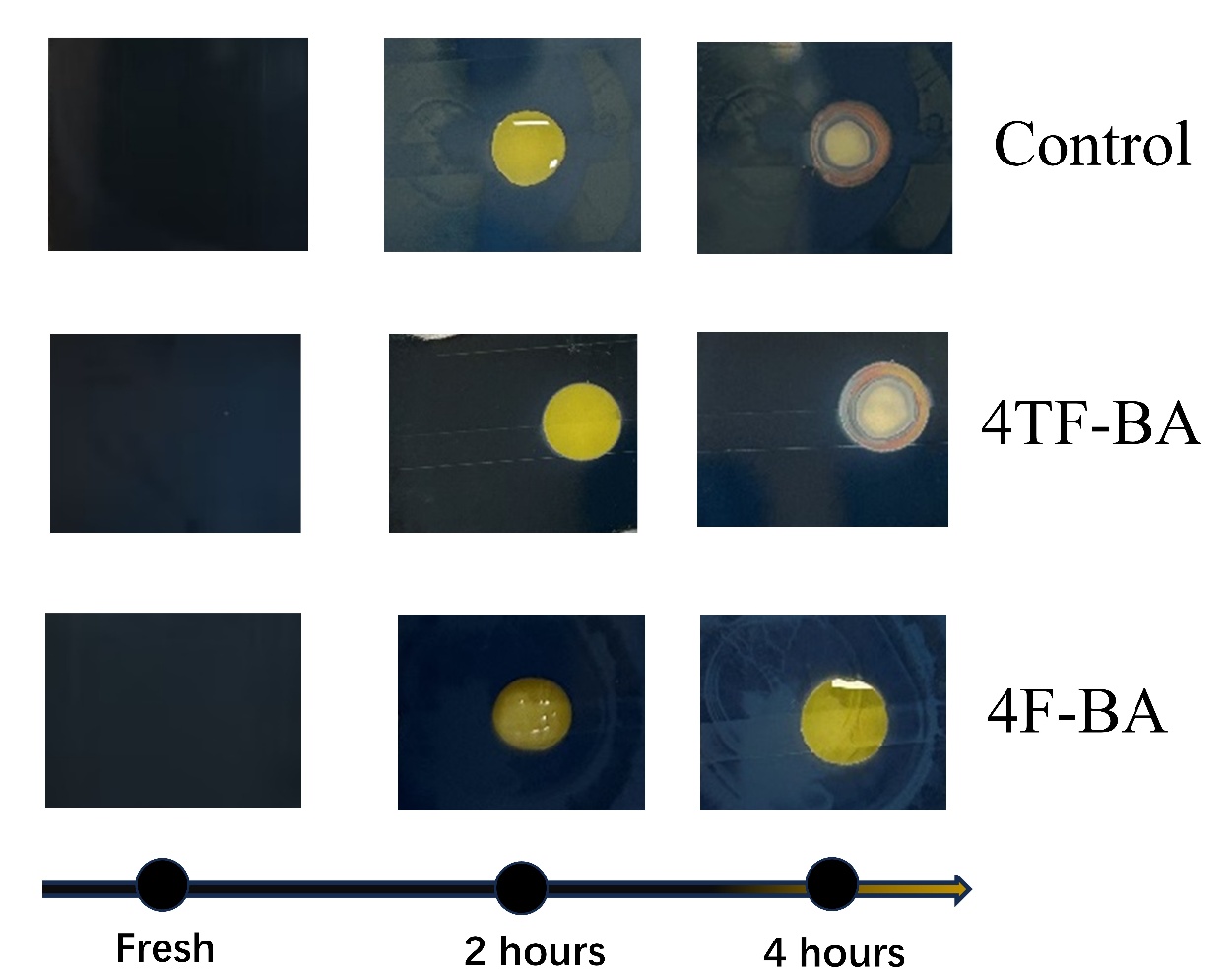


**Fig. S25.** The images of control and modified perovskite films with prolonged water droplet test after storing in air for 4 hours.

**Supporting Tables**

**Table S1.** The fitted carrier lifetime of perovskite films obtained from the TRPL measurements.

| **Device** | **A_1_** | **τ_1_(ns)** | **A_2_** | **τ_2_(ns)** | **τ_ave_(ns)** |
| --- | --- | --- | --- | --- | --- |
| Control | 0.48 | 50.15 | 1.32 | 180.23 | 168.28 |
| 4TF-BA | 0.43 | 98.72 | 1.48 | 443.34 | 422.40 |
| 4F-BA | 0.32 | 129.22 | 1.64 | 682.92 | 663.21 |

**Table S2**. The fitting results of Nyquist impedance by the equivalent circuit.

| **Device** | **R_S_ (Ω)** | **R_rec_ (Ω)** | **C (nF)** |
| --- | --- | --- | --- |
| Control | 37.89 | 2853 | 8.63 |
| 4TF-BA | 24.71 | 4597 | 7.41 |
| 4F-BA | 22.19 | 5462 | 3.04 |

References

1. J. Hafner, Journal of Computational Chemistry **29**, 2044 (2008).

2. Y. Zhao, N. E. Schultz, and D. G. Truhlar, J. Chem. Theory Comput. **2**, 364 (2006).

3. G. Kresse and D. Joubert, Phys. Rev. B **59**, 1758 (1999).

4. S. Kirklin, J. E. Saal, B. Meredig, A. Thompson, J. W. Doak, M. Aykol, S. Rühl, and C. Wolverton, Npj Computational Materials **1**, 15010 (2015).

5. E. Sanville, S. D. Kenny, R. Smith, and G. Henkelman, Journal of Computational Chemistry **28**, 899 (2007).

6. S. Maintz, V. L. Deringer, A. L. Tchougréeff, and R. Dronskowski, Journal of Computational Chemistry **37**, 1030 (2016).
